# Ethnicity Disparities in the Prevalence, Awareness, Treatment, and Control Rates of Hypertension in China

**DOI:** 10.1155/2023/1432727

**Published:** 2023-03-14

**Authors:** Yanan Yang, Kunlin He, Yuewen Zhang, Xiuming Wu, Weizhong Chen, Dongqing Gu, Ziqian Zeng

**Affiliations:** ^1^Department of Epidemiology and Statistics, Chengdu Medical College, Chengdu 610500, China; ^2^Yibin Center for Disease Control and Prevention, Yibin 644000, China; ^3^First Affiliated Hospital, Army Medical University, Chongqing 400038, China

## Abstract

**Objectives:**

Previous studies reported that there were disparities in hypertension management among different ethnic groups, and this study aimed to systematically determine the prevalence, awareness, treatment, and control rates of hypertension in multiple Chinese ethnic groups.

**Methods:**

We searched Embase, PubMed, and Web of Science for articles up to 25 October, 2022. The pooled prevalence, awareness, treatment, and control rates of hypertension were estimated with 95% confidence intervals (CI). The heterogeneity of estimates among studies was assessed by the Cochran *Q* test and *I*^2^ statistic. Meta-regression analyses were conducted to identify the factors influencing the heterogeneity of the pooled prevalence, awareness, treatment, and control rate of hypertension.

**Results:**

In total, 45 publications including 193,788 cases and 587,826 subjects were eligible for the analyses. The lowest prevalence was found in the Han group (27.0%), and the highest prevalence was in the Mongolian population (39.8%). The awareness rates ranged from 24.4% to 58.0% in the four ethnic groups. Both the highest treatment and control rates were found in the Mongolian population (50.6% and 16.0%, respectively), whereas the Yi group had the lowest control rate (8.0%). In addition, the study year, the mean age of subjects, mean body mass index of subjects, tobacco use (%), alcohol use (%), residence (urban%), and education (primary school%) had varied effects on heterogeneity.

**Conclusions:**

These findings highlight the disparities in prevalence, awareness, treatment, and control rates of hypertension in a different ethnic population of China, which could provide suggestions for making targeted prevention measures.

## 1. Introduction

Hypertension is an important risk factor for cardiovascular diseases and other chronic diseases which impose a huge burden on economic and human development globally [1, 2]. Nevertheless, low awareness, treatment, and control of hypertension will handle the prevention progress of cardiovascular diseases [3–5]. In China, the median prevalence of hypertension was 32% in females and 37% in males [6], which were higher than the average level of 20% of women and 24% of men around the world [1]. The awareness, treatment, and control rates reported by a systematic review were only 53%, 44%, and 17%, respectively, among the female population, and these rates were even lower in men [6].

Some studies reported that the prevalence rate of hypertension may be different in each ethnic group, which could be attributed to the different genetic and environmental conditions [7–10]. China is a unified, multinational country, with 56 ethnic groups in all. It is evident that the status and management of hypertension are varied in these groups [11]. Understanding the status of the ethnic groups was beneficial for hypertension control.

Most of the individual studies were conducted for one ethnic group, however, systematically collected data to compare the prevalence, awareness, treatment, and control rates among varied ethnic populations were insufficient. Therefore, this study investigated the disparities in the management of hypertension among ethnic groups to improve hypertension prevention in order to achieve the global noncommunicable disease targets.

## 2. Methods

### 2.1. Data Sources

We searched the three databases (Embase, PubMed, and Web of Science) up to 25 October, 2022. The medical subject heading (MeSH) terms were used for the search strategies. The search strategy was as follows: (blood pressure OR hypertension) AND (epidemiology OR prevalence OR incidence OR awareness OR treatment OR control) AND (China). To maximize the yield of articles, we further screened the references of all included studies or meta-analyses in the database to select additional eligible articles.

### 2.2. Inclusion and Exclusion Criteria

The eligible articles were under the following criteria: (1) articles that were published in peer-review journals in both English and Chinese; (2) articles that defined hypertension as SBP ≥ 140 mmHg or/and DBP ≥ 90 mmHg; or self-reported pharmacological treatment for hypertension; (3) articles that reported the prevalence or awareness or treatment or control rate; (4) articles that provided the cases and sample size or sufficient data to calculate these rates. Some exclusion criteria were set before data extraction: (1) articles that provided limited information; (2) articles that were not the original research articles, such as review, comment, or letter; (3) articles that focused on the treatment or medication of hypertension. Two investigators (G.DQ and Z.ZQ) evaluated each study and checked the eligibility independently.

### 2.3. Data Extraction

Data were extracted by two researchers (Y.YN and Z.ZQ) using the standard information forms, including first author name, publishing year, study year, study design type, ethnicity, case number, sample size, prevalence rate, awareness rate, treatment rate, control rate, mean age of the population, age range of population, mean body mass index (BMI), tobacco use (%), alcohol use (%), gender (female%), residence (residence%), and education (education%). When the data from the same survey were published in more than one publication, we used the largest one.

### 2.4. Quality Assessment

The quality of included studies was independently assessed by two investigators (H. KL and Y. YN) using the adapted Newcastle-Ottawa Scale (NOS) [12]. If there were any disagreements, it was resolved by consultation with another author (G. DQ). Nine items were applied to assess the included studies, and each study received a total score (TS) for methodological quality. The score ranged from 0–10: TS ≥ 7 ranks as “high quality,” 7<TS ≤ 6 ranks as “moderate quality,” and TS < 6 ranks as “low quality.”

### 2.5. Statistical Analysis

A pooled prevalence, awareness, treatment, and control rates of hypertension were estimated with 95% confidence intervals (CI). The heterogeneity of estimates among studies was assessed by the Cochran *Q* test and *I*^2^ statistic. Meta-regression analyses were conducted to identify the factors influencing the heterogeneity. In regression models, the study year (<2010, ≥2010), mean age (years), mean BMI, tobacco use (%), alcohol use (%), gender (female%), residence(urban%), and education (primary school%) were included as covariates. The sensitivity analysis was also conducted to explore the potential heterogeneity. The Egger test and Begg test were performed for publication bias. Statistical analyses were conducted using Stata 14 software (https://www.stata.com/) *p* ≤ 0.05 was defined as statistical significance.

## 3. Results

### 3.1. Characteristics of Included Studies

**Figure 1:**
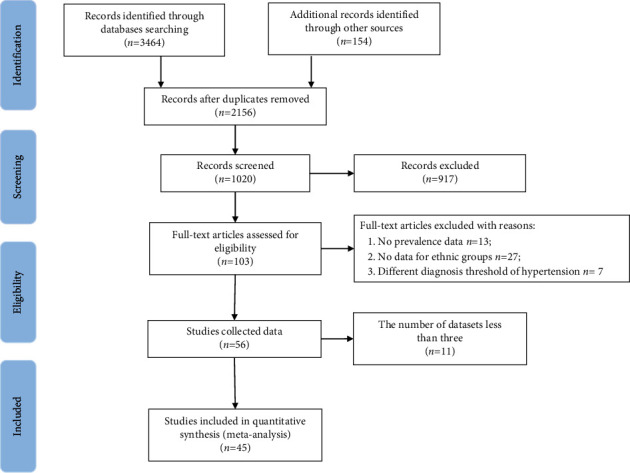
Literature search results.

A PRISMA flowchart was used to illustrate the selection of studies (Figure 1) [13]. We screened a total of 3,618 articles, and finally, 45 publications of 193,788 cases and 587,826 subjects were included in the analyses. According to the number of datasets, a total of four ethnic groups (Han, Tibetan, Yi, and Mongolian) were considered to conduct the meta-analyses. There were 14 datasets used for the Han population, 23 datasets used for the Tibetan population, 7 datasets used for the Yi population, and 10 datasets used for the Mongolian population. There were nine datasets conducted in 1996–2005, 37 datasets investigated in 2006–2015, eight datasets conducted in 2016–2019, and one dataset conducted in 1979. More characteristics of included studies were displayed in Supplementary Table 1.

### 3.2. Quality of the Studies

The quality of included studies was relatively high. According to the results of the quality assessment, 31 (68.9%) studies were categorized as “high quality” and 14 (31.1%) studies were classified as “moderate quality.” The details of the quality assessment are shown in Supplementary Table 2.

### 3.3. Prevalence, Awareness, Treatment, and Control of Hypertension

All prevalence rates were calculated using the cut-off value (140/90 mmHg), and the awareness, treatment, and control rates were calculated among cases with hypertension. The pooled prevalence, awareness, treatment, and control rates of the total population were 31.7%, 42.5%, 33.4%, and 12.2%, respectively. The lowest prevalence rate was found in the Han group (27.0%), and the highest prevalence was in the Mongolian population (39.8%). The awareness rates ranged from 24.4% to 58.0%% in four ethnic groups. The highest treatment and control rates were found in the Mongolian population (50.6% and 16.0%, respectively). The Yi group had the lowest control rate (8.0%), see Table 1.

### 3.4. Results from Meta-Regression Analyses

The meta-regression analyses were conducted for the four ethnic groups, respectively. In the meta-regression model for the Han group, the results of mean age and mean BMI were statistically significant, explaining 40.96% (*p*=0.015) and 35.16% (*p*=0.032) of the heterogeneity in prevalence between studies, respectively. The mean BMI, alcohol use, and study year explained 54.21% (*p*=0.006), 66.83% (*p*=0.001), and 54.20% (*p*=0.003) of the heterogeneity in awareness between studies, respectively. And the mean BMI, alcohol use, study year, and tobacco use were statistically significant, explaining 48.44% (*p*=0.011), 53.19% (*ws*), 40.19% (*p*=0.009), and 26.99% (*p*=0.041) of the heterogeneity in treatment or control between studies (See Table 2). In the meta-regression model for the Mongolian group, the source of heterogeneity for prevalence was mean age (Adj. *R*^2^ = 69.12%, *p*=0.004), and education (Adj. *R*^2^ = 46.36%, *p*=0.039) for control rate (see Table 3). In addition, the residence (Adj. *R*^2^ = 100.00%, *p*=0.048) affected the awareness rate of the Tibetan group (see Supplementary Table 3). The mean BMI (Adj. *R*^2^ = 76.68%, *p*=0.034) and study year (Adj. *R*^2^ = 57.40%, *p*=0.036) also had effects on the prevalence of the Yi group, respectively (see Supplementary Table 4).

### 3.5. Bias and Sensitivity Analysis

31 (68.9%) studies were judged to be of high quality with a low risk of bias, while 14 (31.1%) studies were at moderate risk of bias. In the sensitivity analyses, no individual study was found to have any major impact on the pooled prevalence, awareness, treatment, and control rate of hypertension (see supplementary Figures 1–4). The Egger test was not statistically significant for testing the publication bias, except in analyses of pooled awareness of the Han population (*p*=0.0002) and the pooled treatment of the Tibetan population (*p*=0.0005). The Begg test showed no small-study bias in these meta-analyses, all the *p* values were greater than 0.05.

## 4. Discussion

To our knowledge, this study is the first to systematically summarize the rates of hypertension management in multiple Chinese ethnic groups. In this study, we finally selected four main ethnic groups (Han, Tibetan, Yi, and Mongolian) in our analyses according to the number of databases. Our results indicated that the status varied in these four groups, in which Mongolian had the highest prevalence, treatment, and control rates. However, Yi people had a different story of increasing the prevalence rate and relatively lower the control rate. The results denoted that understanding the differences in varied ethnic groups could inform targeted prevention measures to improve hypertension management and reduce the disparities.

Based on previous findings, the prevalence rates of hypertension would vary in different ethnic populations due to genetic and environmental factors [14, 15]. In our study, the combined prevalence among four ethnic groups was 31.7%, which was slightly higher than the results from the Chinese Hypertension Survey in 2012–2015 which found that the average rate of the Chinese population was 27.9% [16]. In these ethnic groups, the rate of Han nationality was the lowest (27.0%), while the rates of Mongolian and Tibetan were more than 30%. These results were consistent with previous studies [17]. Most of the Tibetan population in China are living in the highlands, and some publications indicated that chronic hypoxia exposure may cause a higher prevalence of hypertension [18]. In addition, according to our previous studies, hypoxia exposure may also increase the risk of other diseases, such as sleep disorders, which were regarded as the risk factors for hypertension [19, 20]. For the Mongolian population, the prevalence was high in the recent thirty years in some large-scale surveys [21]. This phenomenon may be attributable to the changes in their diet culture because Mongolian people prefer to consume meat and milk as the principal food, especially among people living in pastoral regions [22]. Yi population was found to have a low prevalence of both overweight and obesity [23], which may contribute to a lower rate of hypertension (28%). However, the population attribution fraction (PAF) of overweight or obesity in this population was also increasing in recent years. For example, in 1996, the PAF was 27.66% and the rate of hypertension was 5.33% in 1996, while the PAF increased to 33.26% in 2015 with a prevalence of hypertension of 17.2% [24].

Furthermore, among people with hypertension, the awareness, treatment, and control rates were also analyzed in this study. In China, the average rates were 51.5%, 46.1%, and 16.9% reported by a nationwide survey in 2015 [25], which were higher than the rates reported in other developing countries, such as Ghana [26]. In this study, only the rates from the Mongolian population were close to this level, and the Tibetan and Han population slightly lower than that level, while these rates in the Yi group were the lowest. Two possible reasons might account for these results. Firstly, compared to the other groups, Yi people had a relatively lower prevalence of hypertension over a long-term period, which may make them pay less attention to the increase of risk factors at the population level. For example, some studies had reported that Yi people's dietary patterns changed largely in the past two decades, which caused the gradual growth of the prevalence of hypertension [27, 28]. Secondly, the previous studies showed that numerous Yi people migrated from rural to urban areas, which imposed heavy pressure on the prevention of these two distinct groups. Yi migrants usually had a higher prevalence of hypertension than Yi farmers, and it was useless to implement the same measures to improve their awareness, treatment, and control situation among Yi people. More targeted strategies and policies are needed in the Yi population.

There are some strengths in our study. The pooled estimates of the prevalence, awareness, treatment, and control of hypertension in four main Chinese ethnic groups were reported comprehensively. The search strategy involved plenty of studies and subjects based on the standard of PRISMA [29]. Our results were robust and corroborated by further sensitivity analyses and the tests of small-study bias and publication bias. Nonetheless, there are also some limitations. Firstly, high heterogeneity was found in our meta-analyses, which were familiar with other meta-analytic studies focusing on the pooled rates because of the differences in the study population, inclusion and exclusion criteria, and assessment [30, 31]. In this systematic review, we also included some common covariates to explore the source of heterogeneity, such as BMI, gender, tobacco use, alcohol use, age, study year, and education. Of which, mean age, mean BMI, study year, alcohol use, and tobacco use were significantly associated with the rates of hypertension management. Secondly, some other Chinese ethnic groups, such as Zhuang, Uygur, and Hui, were not included in the meta-analyses because of insufficient datasets.

## 5. Conclusions

The systematic review included four main Chinese ethnic groups and conducted meta-analyses for prevalence, awareness, treatment, and control rates of hypertension. The findings suggest that hypertension management varied in these Chinese ethnic groups which Mongolian and Tibetan had higher prevalence rates. Notably, the Yi population was regarded as the low-risk population for hypertension, and it should pay more attention to prevention because the prevalence rates were rising in recent years, while their awareness, treatment, and control rates were still low. We call for further studies to better understand the specific risk factors and mechanisms of hypertension in the Tibetan and Mongolian populations to make more targeted preventive measures. In addition, for Yi people, it is urgent to implement interventions to improve their awareness and treatment, and finally achieve the goal of hypertension control.

## Figures and Tables

**Table 1:** Prevalence, awareness, treatment, and control of hypertension by survey year, ethnicity groups.

Groups		Number of datasets	Number of participants	Min (%)	Max (%)	Pooled rate (%) (95% CI)	*I* ^2^ (%)
Total population	Prevalence	54	587,826	4.9	62.4	31.7 (28.4–35.0)	99.8
	Awareness	34	510,150	6.0	83.0	42.5 (37.2–47.8)	99.8
	Treatment	35	525,106	2.6	89.1	33.4 (28.8–37.9)	99.7
	Control	35	525,106	0.3	43.6	12.2 (9.2–15.2)	99.8

Han population	Prevalence	14	492,498	16.0	56.0	27.0 (20.8–33.3)	99.9
	Awareness	13	461,315	19.5	66.5	41.3 (35.5–47.0)	95.8
	Treatment	14	476,271	13.0	53.3	31.6 (26.3–36.9)	96.4
	Control	14	476,271	1.2	26.7	12.7 (7.2–18.2)	97.9

Tibetan population	Prevalence	23	46,492	11.2	62.4	32.1 (25.9–38.4)	99.6
	Awareness	9	16,110	6.0	69.4	35.1 (18.3–51.8)	99.6
	Treatment	9	16,110	2.6	59.1	22.3 (13.4–31.2)	99.0
	Control	9	16,110	0.3	31.8	8.9 (5.2–12.5)	98.4

Mongolian population	Prevalence	10	33,914	25.4	53.0	39.8 (34.5–45.2)	99.0
	Awareness	9	29,387	29.7	83.0	58.0 (43.3–72.6)	99.7
	Treatment	9	29,387	23.6	89.1	50.6 (36.7–64.6)	99.6
	Control	9	29,387	0.7	43.6	16.0 (9.3–22.6)	99.5

Yi population	Prevalence	7	25,393	10.4	47.5	28.0 (17.8–38.2)	99.5
	Awareness	3	3,463	13.6	35.0	24.4 (14.1–34.6)	97.8
	Treatment	3	3,463	10.6	31.1	23.0 (10.7–35.4)	98.6
	Control	3	3,463	7.2	9.1	8.0 (6.7–9.2)	35.5

**Table 2:** Meta-regression analysis of the Han group to explore potential sources of heterogeneity, prevalence, awareness, treatment, and control.

Variable	N	Coefficient	Tau^2^	Adj. *R*^2^(%)	*I* ^2^	*p*
*Prevalence*						
Block 0	14	0.2702878	0.01206		99.93	<0.001
Age (mean)	12	0.0127801	0.00726	40.96	99.90	0.015
BMI (mean)	11	0.0617196	0.00876	35.16	99.89	0.032
Tobacco use (%)	13	0.0003943	0.01286	−9.08	99.94	0.937
Alcohol use (%)	13	−0.0016196	0.01241	−5.27	99.94	0.545
Gender (women%)	12	0.0143087	0.00880	23.25	99.89	0.065
Education (primary %)	9	0.0005281	0.01571	−14.09	99.96	0.909
Residence (urban%)	6	0.0014385	0.01998	−23.07	99.97	0.814
Study year	14		0.01178	2.31	99.94	
<2010 (ref)	9	0.0693379				0.275
≥2010	5					

*Awareness*						
Block 0	13	0.4096955	0.02899		99.73	<0.001
Age (mean)	11	0.0048229	0.03635	−7.20	99.78	0.572
BMI (mean)	11	0.1053923	0.01553	54.21	99.75	0.006
Tobacco use (%)	12	−0.0157154	0.02349	23.61	98.68	0.065
Alcohol use (%)	12	−0.0119039	0.01020	66.83	99.63	0.001
Gender (women %)	11	−0.0092420	0.03079	−5.51	99.79	0.501
Education (primary %)	9	0.0071913	0.01629	−4.04	99.78	0.418
Residence (urban %)	6	−0.0048421	0.00487	19.24	98.83	0.222
Study year	13		0.01328	54.20	99.58	
<2010	8	−0.2562017				0.003
≥2010 (ref)	5					

*Treatment*						
Block 0	14	0.3145307	0.02357		99.71	<0.001
Age (mean)	12	0.0053511	0.02558	−3.53	99.76	0.445
BMI (mean)	11	0.0902302	0.01404	48.44	99.75	0.011
Tobacco use (%)	13	−0.0116078	0.01952	13.93	99.21	0.117
Alcohol use (%)	13	−0.0091629	0.01062	53.19	99.61	0.003
Gender (women %)	12	−0.0065091	0.02323	−6.64	99.76	0.576
Education (primary %)	10	0.0012871	0.01655	−11.78	99.76	0.785
Residence (urban %)	7	−0.0029320	0.00320	10.91	98.87	0.262
Study year	14		0.01410	40.19	99.55	
<2010	8	−0.2010464				0.009
≥2010 (ref)	6					

*Control*						
Block 0	14	0.1274783	0.00518		99.91	<0.001
Age (mean)	12	0.0016195	0.00547	−7.16	99.93	0.618
BMI (mean)	11	0.0285270	0.00440	17.78	99.87	0.106
Tobacco use (%)	13	−0.0066053	0.00343	26.99	99.18	0.041
Alcohol use (%)	13	−0.0024945	0.00413	12.04	99.86	0.132
Gender (women %)	12	−0.0026796	0.00538	−7.47	99.90	0.634
Education (primary %)	10	0.0000799	0.00740	−12.56	99.93	0.980
Residence (urban %)	7	−0.0018121	0.00193	2.33	98.12	0.359
Study year	14		0.00443	14.41	99.65	
<2010	8	−0.0654570				0.098
≥2010 (ref)	6					

**Table 3:** Meta-regression analysis of the Mongolian group to explore potential sources of heterogeneity, prevalence, awareness, treatment, and control.

Variable	*N*	Coefficient	Tau^2^	Adj. *R*^2^(%)	*I* ^*2*^	*p*
*Prevalence*						
Block 0	10	0.3981608	0.00907		99.01	<0.001
Age (mean)	9	0.0114007	0.00228	69.12	97.12	0.004
BMI (mean)	7	0.0111736	0.00661	−9.59	98.93	0.523
Tobacco use (%)	9	0.0015801	0.00820	−11.13	99.11	0.658
Alcohol use (%)	9	−0.0000628	0.00844	−14.36	99.10	0.987
Gender (women %)	10	0.0073648	0.00914	−0.77	98.91	0.365
Education (primary %)	8	0.0033455	0.00737	3.84	99.01	0.300
Residence (urban %)	9	0.0027103	0.00866	14.78	99.07	0.167
Study year			0.00911	−4.64	97.63	
≥2010	6	−0.0520017				0.430
<2010 (ref)	4					

*Awareness*						
Block 0	9	0.5795011	0.02745		99.68	<0.001
Age (mean)	8	0.0071019	0.03356	−7.80	99.62	0.513
BMI (mean)	6	0.0420745	0.02073	16.46	99.38	0.232
Tobacco use (%)	8	−0.0040465	0.03478	−11.71	99.75	0.623
Alcohol use (%)	8	−0.0001928	0.03628	−16.52	99.75	0.980
Gender (women %)	9	0.0145477	0.02756	−0.40	99.64	0.360
Education (primary %)	8	−0.0037034	0.03433	−10.26	99.71	0.580
Residence (urban %)	8	0.0018788	0.01985	−7.70	99.22	0.509
Study year			0.02878	−4.87	99.71	
<2010	3	−0.0957634				0.453
≥2010 (ref)	6					

*Treatment*						
Block 0	9	0.5063089	0.04006		99.65	<0.001
Age(mean)	8	0.0001872	0.05213	−16.74	99.72	0.989
BMI (mean)	6	0.0373971	0.01717	15.08	99.30	0.242
Tobacco use (%)	8	−0.0017870	0.05182	−16.06	99.71	0.857
Alcohol use (%)	8	0.0112595	0.03857	13.63	99.71	0.197
Gender (women %)	9	0.0318632	0.02748	31.40	99.43	0.068
Education (primary %)	8	−0.0118833	0.03174	28.92	99.57	0.098
Residence (urban %)	8	0.0031071	0.03512	−3.48	99.42	0.414
Study year			0.04137	−3.26	99.65	
<2010	3	−0.1244581				0.417
≥2010 (ref)	6					

*Control*						
Block 0	9	0.1601280	0.01308		99.45	0.003
Age (mean)	8	−0.0037464	0.01698	−13.03	99.51	0.628
BMI (mean)	6	0.0239634	0.00244	47.00	99.30	0.081
Tobacco use (%)	8	−0.0023905	0.01709	−13.79	98.79	0.681
Alcohol use (%)	8	0.0066254	0.01318	12.24	99.45	0.202
Gender (women %)	9	0.0164198	0.01016	22.37	98.30	0.117
Education (primary %)	8	−0.0083389	0.00806	46.36	99.44	0.039
Residence (urban %)	8	0.0129200	0.00062	−16.83	97.71	0.788
Study year			0.01045	20.09	98.45	
<2010	3	−0.1244728				0.136
≥2010 (ref)	6					

## Data Availability

The data used to support the findings of this research are available from major databases, including Embase, PubMed, and Web of Science.

## Supplementary Materials

Supplementary Materials Supplementary Table 1: the characteristics of included studies. Supplementary Table 2: the quality of references assessed by the adjusted Newcastle Ottawa Assessment Scale. Supplementary Table 3: meta-regression analysis of the Tibetan group to explore potential sources of heterogeneity, prevalence, awareness, treatment, and control. Supplementary Table 4: meta-regression analysis of the Yi group to explore potential sources of heterogeneity and prevalence. Supplementary Figure 1: the sensitivity analyses of data from Han population, 1A prevalence of Han, 1B awareness of Han, 1C treatment of Han, and 1D control of Han. Supplementary Figure 2: the sensitivity analyses of data from Tibetan population, 2A prevalence of Tibetan, 2B awareness of Tibetan, 2C treatment of Tibetan, and 2D control of Tibetan. Supplementary Figure 3: the sensitivity analyses of data from Yi population, 3A prevalence of Yi, 3B awareness of Y, 3C treatment of Y, and 3D Control of Yi. Supplementary Figure 4: the sensitivity analyses of data from Mongolian population, 4A prevalence of Mongolian, 4B awareness of Mongolian, 4C treatment of Mongolian and 4D control of Mongolian.
