# Supplementary material for: Ethnicity Disparities in the Prevalence, Awareness, Treatment, and Control Rates of Hypertension in China
Source: Int J Hypertens. 2023 Mar 14;2023:1432727. doi: 10.1155/2023/1432727 (PMC10030218; doi:10.1155/2023/1432727)
Supplement: Supplementary Materials — Supplementary Table 1: the characteristics of included studies. Supplementary Table 2: the quality of references assessed by the adjusted Newcastle Ottawa Assessment Scale. Supplementary Table 3: meta-regression analysis of the Tibetan group to explore potential sources of heterogeneity, prevalence, awareness, treatment, and control. Supplementary Table 4: meta-regression analysis of the Yi group to explore potential sources of heterogeneity and prevalence. Supplementary Figure 1: the sensitivity analyses of data from Han population, 1A prevalence of Han, 1B awareness of Han, 1C treatment of Han, and 1D control of Han. Supplementary Figure 2: the sensitivity analyses of data from Tibetan population, 2A prevalence of Tibetan, 2B awareness of Tibetan, 2C treatment of Tibetan, and 2D control of Tibetan. Supplementary Figure 3: the sensitivity analyses of data from Yi population, 3A prevalence of Yi, 3B awareness of Y, 3C treatment of Y, and 3D Control of Yi. Supplementary Figure 4: the sensitivity analyses of data from Mongolian population, 4A prevalence of Mongolian, 4B awareness of Mongolian, 4C treatment of Mongolian and 4D control of Mongolian. [file 1432727.f1.docx]

**Supplementary Table 1. The characteristics of included studies**

| **First author** | **Publishing year** | **Study design** | | **Ethnicity** | | **Cases** | | **Sample size** | | **Awareness rate (%)** | | **Treatment rate (%)** | | **Control rate(%)** | | **Prevalence rate(%)** | | **Survey year** | | **Age range(y)** | | **Mean age(y)** | | **BMI(Kg/m2)** |  |
| --- | --- | --- | --- | --- | --- | --- | --- | --- | --- | --- | --- | --- | --- | --- | --- | --- | --- | --- | --- | --- | --- | --- | --- | --- | --- |
| Wei,J | 2021 | Cross-sectional | Han | | 3579 | |  | | 56.7 | | 47.5 | | 21.3 | |  | | 2011 | | >=18 | | 60±12.4 | | 25.3±5.4 | | |
| Congyi Zheng | 2020 | Cross-sectional | Han | | 84470 | | 299220 | | 51.39 | | 45.63 | | 16.23 | | 28.23 | | 2012-2015 | | >=18 | | 48.2(48.2-48.3) | | 23.81(22.47-22.77) | | |
| Mulalibieke Heizhati | 2020 | Cross-sectional | Han | | 566 | | 2709 | | 55.5 | | 41.5 | | 14.5 | | 20.9 | | 2014-2015 | | >=18 | |  | |  | | |
| Lin Wang | 2019 | Cross-sectional | Han | | 4450 | | 19180 | | 66.5 | | 47 | | 17.5 | | 23.2 | | 2014-2016 | | >=18 | | 44.0±15.3 | | 24.5±3.4 | | |
| Lei Zhao | 2018 | Cross-sectional | Han | | 4337 | | 14956 | |  | | 31.7 | | 8.8 | | 29 | | 2014-2015 | | 15-97 | | 41.55±16.55 | |  | | |
| Yin Ruixing | 2008 | Cross-sectional | Han | | 193 | | 1173 | | 21.76 | | 12.95 | | 8.29 | | 16.45 | | 2002 | | 15-89 | | 38.29±16.84 | | 22.15±2.94 | | |
| Zhaoqing Sun | 2008 | Cross-sectional | Han | | 13269 | | 36154 | | 29.2 | | 23.5 | | 1.2 | | 36.7 | | 2004-2006 | | >=35 | | 51.2 ± 11.8 | | 23.1 ± 3.1 | | |
| Yin Ruixing | 2006 | Cross-sectional | Han | | 152 | | 520 | | 19.5 | | 13.3 | | 9.4 | | 29.2 | |  | | 40-89 | | 55.8±11.3 | | 22.4±2.6 | | |
| Yin Ruixing | 2006 | Cross-sectional | Han | | 163 | | 1018 | | 20.9 | | 15.3 | | 10.4 | | 16 | | 2003 | | 6-89 | | 42.95±17.11 | | 22.22±2.62 | | |
| Yi Zhao | 2010 | Cross-sectional | Han | | 250 | | 1655 | | 31.5 | | 13.6 | | 2.7 | | 15.1 | | 2008-2009 | | >=25 | |  | |  | | |
| Tao Xu | 2009 | Cross-sectional | Han | | 7986 | | 26002 | | 59.3 | | 53.3 | | 26.7 | | 30.7 | | 2007 | | >=18 | | 40.3 ± 11.4 | | 22.7 ± 3.1 | | |
| Zhaoqing Sun | 2010 | Cross-sectional | Han | | 4803 | | 19806 | |  | |  | |  | | 24.25 | | 2004-2006 | | >=35 | |  | |  | | |
| Huang F | 2008 | Cross-sectional | Han | | 197 | | 1173 | | 21.76 | | 12.95 | | 8.29 | | 16.79 | |  | | >=15 | | 38.3± 16.8 | | 22.15± 2.94 | | |
| Li Cai | 2012 | Cross-sectional | Han | | 2045 | | 5760 | | 42.5 | | 35.9 | | 11.8 | | 35.5 | | 2008 | | 18-79 | |  | |  | | |
| Xu X | 2021 | Cross-sectional | Han | | 35376 | | 63172 | | 53.6 | | 44.2 | | 20.2 | | 56 | | 2015 | | 35-75 | | 54.4±9.4 | | 25.8±3.6 | | |
| Xingshan Zhao | 2012 | Cross-sectional | Tibetan | | 392 | | 701 | | 19.9 | | 2.6 | | 0.3 | | 55.9 | | 2008-2009 | | 40-89 | | 55.4±11.7 | | 22.8±3.0 | | |
| Le Yue | 2016 | Cross-sectional | Tibetan | | 208 | | 1000 | |  | |  | |  | | 20.8 | | 2015 | |  | | 46.0±15.5 | | 23.3±3.0 | | |
| Rendan Deng | 2020 | Cross-sectional | Tibetan | | 1511 | | 10493 | |  | |  | |  | | 14.4 | | 2018 | | >=15 | |  | | 44.1±15.7 | | |
| Xiao fei Huang | 2019 | Cross-sectional | Tibetan | | 1501 | | 5053 | |  | |  | |  | | 29.7 | | 2013 | | >=18 | | 41.8±13.6 | | 23.6±3.7 | | |
| Lhamo Y. Sherpa | 2013 | Cross-sectional | Tibetan | | 256 | | 692 | | 69.4 | | 59.1 | | 19.5 | | 37 | | 2010 | | 30-80 | |  | |  | | |
| Ci Song | 2020 | Cross-sectional | Tibetan | | 658 | | 1631 | |  | |  | |  | | 40.6 | | 2017 | | 20-80 | |  | | 23.7(21.3,26.4) | | |
| Yang Shen | 2017 | Cross-sectional | Tibetan | | 1180 | | 4198 | | 35 | | 31.1 | | 8.7 | | 26.7 | | 2013 | | 20-59 | | 39.77±8.92 | | 24.24±3.26 | | |
| Xi Zheng | 2012 | Cross-sectional | Tibetan | | 701 | | 1370 | | 63.5 | | 24.3 | | 7.7 | | 51.2 | | 2010 | | >=18 | |  | |  | | |
| Kozo Matsubayashi | 2009 | Cross-sectional | Tibetan | | 35 | | 97 | |  | |  | |  | | 36 | | 2008 | | >=60 | | 66.7±5.1 | |  | | |
| Longjian Liu | 2001 | Cross-sectional | Tibetan | | 50 | | 125 | |  | |  | |  | | 39.7 | | 2000 | | 48-56 | |  | | 20.7±0.4 | | |
| Shin Fu Sun | 1986 | Cross-sectional | Tibetan | | 409 | | 3640 | |  | |  | |  | | 11.24 | | 1979 | | >=15 | |  | |  | | |
| Li T | 2021 | Cross-sectional | Tibetan | | 548 | | 2228 | | 32.3 | | 21.7 | | 6.2 | | 24.6 | | 2018 | | 18-80 | | 45.00±14.01 | | 23.55±2.86 | | |
| Sun P | 2019 | Cross-sectional | Tibetan | | 401 | | 1486 | |  | |  | |  | | 27.0 | | 2018 | | >=18 | |  | | 24.74±4.03 | | |
| Liu K | 2018 | Cross-sectional | Tibetan | | 115 | | 594 | |  | |  | |  | | 19.4 | | 2013-2014 | | >=18 | | 41.9±15.5 | | 25.3±4.19 | | |
| Meng Q | 2018 | Cross-sectional | Tibetan | | 68 | | 1384 | |  | |  | |  | | 4.9 | | 2016-2017 | | >=18 | | 45.1±15.6 | | 24.7±4.2 | | |
| Meng Q | 2018 | Cross-sectional | Tibetan | | 68 | | 798 | |  | |  | |  | | 8.5 | | 2016-2017 | | >=18 | | 45.8±16.7 | | 24.4±4.0 | | |
| Okumiya K | 2016 | Cross-sectional | Tibetan | | 508 | | 1258 | |  | |  | |  | | 40.4 | | 2008-2011 | | 40-87 | | 58.0±11.5 | | 24.4±4.5 | | |
| Xu S | 2015 | Cross-sectional | Tibetan | | 1035 | | 1659 | |  | |  | |  | | 62.4 | | 2010-2011 | | >=18 | | 44.0±15.2 | |  | | |
| Chen W | 2011 | Cross-sectional | Tibetan | | 500 | | 1289 | |  | |  | |  | | 38.8 | | 2010 | | >=18 | | 43.7±14.3 | | 24.3±4.1 | | |
| Li X | 2015 | Cross-sectional | Tibetan | | 355 | | 1042 | | 16.9 | | 13.2 | | 4.5 | | 34.1 | | 2015 | | 18-70 | | 42.00±8.84 | | 23.86±0.28 | | |
| Li X | 2015 | Cross-sectional | Tibetan | | 190 | | 984 | | 9.5 | | 4.2 | | 1.6 | | 19.3 | | 2015 | | 18-70 | | 41.65±9.22 | | 22.17±0.14 | | |
| Huang X | 2016 | Cross-sectional | Tibetan | | 1554 | | 3400 | | 5.98 | | 21.86 | | 2.95 | | 45.7 | | 2013-2014 | | 15-92 | |  | |  | | |
| Zheng X | 2010 | Cross-sectional | Tibetan | | 701 | | 1370 | | 63.5 | | 24.3 | | 31.8 | | 51.2 | | 2010 | | >=18 | |  | |  | | |
| Mulalibieke Heizhati | 2020 | Cross-sectional | Mongolian | | 195 | | 769 | | 55.1 | | 42.4 | | 16.1 | | 25.3 | | 2014-2015 | | >=18 | |  | |  | | |
| Lin Wang | 2019 | Cross-sectional | Mongolian | | 609 | | 2189 | | 65.3 | | 45.1 | | 14.8 | | 27.8 | | 2014-2016 | | >=15 | | 44.0±15.3 | | 24.5±3.4 | | |
| Zhaoqing Sun | 2008 | Cross-sectional | Mongolian | | 3879 | | 9236 | | 29.7 | | 23.6 | | 0.7 | | 42 | | 2004-2006 | | >=35 | | 51.2±11.8 | | 23.6±3.1 | | |
| Zhaoqing Sun | 2010 | Cross-sectional | Mongolian | | 1569 | | 4554 | |  | |  | |  | | 34.45 | | 2004-2006 | | >=35 | |  | |  | | |
| H Gu | 2015 | Cross-sectional | Mongolian | | 871 | | 2035 | | 42 | | 35.9 | | 5.1 | | 42.8 | | 2005-2007 | | 35-70 | | 48.3±8.7 | | 20.6±4.1 | | |
| Zhang CY | 2012 | Cross-sectional | Mongolian | | 1726 | | 3259 | | 83.03 | | 67.57 | | 17.5 | | 52.96 | | 2008-2009 | | >=55 | | 65.6±7.5 | |  | | |
| Guoju Li | 2016 | Cross-sectional | Mongolian | | 291 | | 925 | | 69.07 | | 89.05 | | 43.58 | | 31.46 | | 2014 | | 20-80 | | 44.43±13.47 | |  | | |
| Peiyao Yu | 2021 | Cross-sectional | Mongolian | | 1086 | | 2426 | | 66.48 | | 58.93 | | 16.48 | | 44.77 | | 2018-2020 | | >=18 | | 50.68±14.00 | | 26.19±7.82 | | |
| Yu P | 2021 | Cross-sectional | Mongolian | | 1086 | | 2462 | | 66.48 | | 58.93 | | 16.48 | | 44.11 | | 2018-2020 | |  | | 50.68±14.00 | | 26.19±7.82 | | |
| Xu X | 2021 | Cross-sectional | Mongolian | | 3165 | | 6086 | | 44.6 | | 34.2 | | 15 | | 52.7 | | 2015 | | 35-75 | | 54.4±9.4 | | 25.8±3.6 | | |
| Jia Zhang | 2020 | Cross-sectional | Yi | | 328 | | 1481 | |  | |  | |  | | 5.33 | | 1996 | |  | |  | |  | | |
| Jia Zhang | 2020 | Cross-sectional | Yi | | 1084 | | 3664 | |  | |  | |  | | 9.06 | | 2007-2008 | |  | |  | |  | | |
| Jia Zhang | 2020 | Cross-sectional | Yi | | 1552 | | 3267 | |  | |  | |  | | 17.2 | | 2015 | |  | |  | |  | | |
| Y Gao | 2014 | Cross-sectional | Yi | | 206 | | 621 | | 35 | | 31.1 | | 8.7 | | 33.2 | | 2007 | | >=20 | | 44.6±14.7 | | 24.0±3.5 | | |
| Y Gao | 2014 | Cross-sectional | Yi | | 66 | | 634 | | 13.6 | | 10.6 | | 9.1 | | 10.4 | | 2007 | |  | | 45.1±14.2 | | 21.1±3.1 | | |
| Zhi-li Yang | 2004 | Cross-sectional | Yi | | 445 | | 3020 | |  | |  | |  | | 14.74 | | 2004 | | >=15 | |  | |  | | |
| Lixing Chen | 2015 | Cross-sectional | Yi | | 850 | | 2208 | | 24.8 | | 27.5 | | 7.2 | | 37 | | 2012 | | >=50 | |  | |  | | |

**Supplementary Table 2. The quality of references assessed by the adjusted Newcastle Ottawa assessment Scale**

| First Author | 1) Define the source of information (survey, record review) | 2) List inclusion and exclusion criteria for exposed and unexposed subjects (cases and controls) or refer to previous publications | 3) Indicate time period used for identifying patients | 4) Indicate whether or not subjects were consecutive if not population-based | 5) Indicate if evaluators of subjective components of study were masked to other aspects of the status of the participants | 6) Describe any assessments undertaken for quality assurance purposes (e.g., test/retest of primary outcome measurements) | 7) Explain any patient exclusions from analysis | 8) Describe how confounding was assessed and/or controlled. | 9) If applicable, explain how missing data were handled in the analysis | 10) Summarize patient response rates and completeness of data collection | 11) Clarify what follow-up, if any, was expected and the percentage of patients for which incomplete data or follow-up was obtained | Total score | Ranking |
| --- | --- | --- | --- | --- | --- | --- | --- | --- | --- | --- | --- | --- | --- |
| Wei,J | 1 | 1 | 1 | 1 | 1 | 1 | 0 | 1 | 1 | 0 | 0 | 8 | High |
| Congyi Zheng | 1 | 1 | 1 | 1 | 0 | 1 | 1 | 1 | 1 | 1 | 1 | 10 | High |
| Mulalibieke Heizhati | 1 | 1 | 1 | 1 | 0 | 1 | 0 | 1 | 0 | 1 | 1 | 8 | High |
| Lin Wang | 1 | 1 | 1 | 1 | 0 | 1 | 1 | 1 | 1 | 1 | 1 | 10 | High |
| Lei zhao | 1 | 1 | 1 | 1 | 1 | 0 | 0 | 0 | 0 | 1 | 0 | 6 | Moderate |
| Yinrui Xing | 1 | 1 | 0 | 1 | 1 | 1 | 1 | 0 | 0 | 1 | 0 | 7 | Moderate |
| Zhaoqing Sun | 1 | 1 | 1 | 1 | 1 | 1 | 1 | 0 | 0 | 1 | 0 | 8 | High |
| Yin Ruixing | 1 | 1 | 1 | 1 | 1 | 1 | 1 | 1 | 0 | 0 | 0 | 8 | High |
| Yin Ruixing | 1 | 1 | 1 | 1 | 1 | 1 | 1 | 0 | 0 | 0 | 0 | 7 | Moderate |
| Yi Zhao | 1 | 1 | 1 | 1 | 1 | 1 | 0 | 1 | 0 | 1 | 1 | 9 | High |
| Tao Xu | 1 | 1 | 1 | 1 | 1 | 1 | 0 | 1 | 0 | 1 | 1 | 9 | High |
| Zhaoqing Sun | 1 | 1 | 1 | 1 | 1 | 1 | 0 | 1 | 0 | 1 | 1 | 9 | High |
| Huang F | 1 | 1 | 0 | 1 | 1 | 1 | 0 | 0 | 0 | 0 | 1 | 6 | Moderate |
| Li Cai | 1 | 1 | 1 | 1 | 0 | 1 | 1 | 1 | 1 | 1 | 0 | 9 | High |
| Xingshan Zhao | 1 | 1 | 1 | 1 | 0 | 1 | 1 | 1 | 1 | 1 | 1 | 10 | High |
| Le Yue | 1 | 1 | 1 | 1 | 1 | 1 | 1 | 1 | 0 | 1 | 0 | 9 | High |
| Rendan Deng | 1 | 1 | 1 | 1 | 1 | 1 | 0 | 1 | 0 | 1 | 0 | 8 | High |
| Xiao fei Huang | 1 | 0 | 1 | 1 | 1 | 1 | 1 | 0 | 0 | 1 | 1 | 8 | High |
| Lhamo Y. Sherpa | 1 | 1 | 1 | 1 | 1 | 1 | 0 | 1 | 0 | 1 | 0 | 8 | High |
| Ci Song | 1 | 1 | 1 | 1 | 1 | 1 | 1 | 1 | 0 | 0 | 0 | 8 | High |
| Yang Shen | 1 | 1 | 1 | 1 | 1 | 1 | 1 | 1 | 0 | 1 | 0 | 9 | High |
| Xi Zheng | 1 | 0 | 1 | 1 | 1 | 0 | 0 | 1 | 0 | 0 | 0 | 6 | Moderate |
| Kozo Matsubayashi | 1 | 1 | 1 | 1 | 1 | 0 | 0 | 1 | 0 | 1 | 1 | 8 | High |
| Longjian Liu | 1 | 1 | 1 | 1 | 1 | 1 | 0 | 1 | 0 | 0 | 0 | 7 | Moderate |
| Shin Fu Sun | 1 | 1 | 1 | 1 | 1 | 1 | 0 | 1 | 0 | 1 | 0 | 8 | High |
| Jia Zhang | 1 | 0 | 1 | 1 | 1 | 1 | 0 | 1 | 1 | 1 | 1 | 9 | High |
| Y Gao | 1 | 1 | 1 | 1 | 1 | 1 | 1 | 1 | 0 | 0 | 0 | 8 | High |
| Zhi-li Yang | 1 | 1 | 0 | 1 | 1 | 0 | 0 | 0 | 0 | 0 | 0 | 6 | Moderate |
| Lixing Chen | 1 | 1 | 1 | 1 | 1 | 1 | 1 | 1 | 0 | 0 | 0 | 8 | High |
| H Gu | 1 | 0 | 1 | 1 | 1 | 1 | 0 | 1 | 0 | 0 | 0 | 6 | Moderate |
| Zhang CY | 1 | 1 | 1 | 1 | 1 | 1 | 0 | 1 | 0 | 1 | 0 | 8 | High |
| Guoju Li | 1 | 1 | 1 | 1 | 1 | 1 | 1 | 1 | 0 | 0 | 0 | 8 | High |
| Peiyao Yu | 1 | 1 | 1 | 1 | 1 | 1 | 0 | 1 | 0 | 0 | 0 | 7 | Moderate |
| Li T | 1 | 1 | 1 | 1 | 1 | 1 | 1 | 0 | 0 | 1 | 0 | 8 | High |
| Xu X | 1 | 1 | 1 | 0 | 0 | 1 | 0 | 1 | 0 | 1 | 0 | 6 | Moderate |
| Yu P | 1 | 1 | 1 | 1 | 0 | 1 | 1 | 1 | 0 | 1 | 0 | 8 | High |
| Sun P | 1 | 1 | 0 | 0 | 1 | 1 | 1 | 1 | 0 | 1 | 0 | 7 | Moderate |
| Liu K | 1 | 1 | 1 | 1 | 1 | 1 | 1 | 1 | 0 | 0 | 0 | 8 | High |
| Meng Q | 1 | 1 | 1 | 1 | 1 | 1 | 0 | 1 | 0 | 1 | 0 | 8 | High |
| Okumiya K | 1 | 1 | 1 | 0 | 0 | 1 | 1 | 1 | 0 | 1 | 0 | 7 | Moderate |
| Xu S | 1 | 1 | 1 | 1 | 1 | 1 | 1 | 1 | 0 | 1 | 0 | 9 | High |
| Chen W | 1 | 1 | 0 | 0 | 1 | 1 | 0 | 1 | 0 | 1 | 0 | 6 | Moderate |
| Li X | 1 | 1 | 0 | 0 | 0 | 1 | 1 | 1 | 0 | 1 | 0 | 6 | Moderate |
| Huang X | 1 | 1 | 1 | 1 | 1 | 1 | 1 | 1 | 0 | 0 | 0 | 8 | High |
| Zheng X | 1 | 1 | 1 | 1 | 1 | 1 | 1 | 1 | 0 | 0 | 0 | 8 | High |

**Table 3** **Meta-regression analysis of the Tibetan group to explore potential sources of heterogeneity, prevalence, awareness, treatment, control**

| Variable | Prevalence | | | | | | Variable | Awareness | | | | | |
| --- | --- | --- | --- | --- | --- | --- | --- | --- | --- | --- | --- | --- | --- |
|  | N | Coefficient | Tau^2^ | Adj.R^2^(%) | *I^2^* | *P* |  | N | Coefficient | Tau^2^ | Adj.R^2^(%) | *I^2^* | *P* |
| Block 0 | 23 | 0.3214975 | 0.02401 |  | 99.64 | <0.001 | Block 0 | 9 | 0.3507229 | 0.06032 |  | 99.63 | 0.003 |
| Age(mean) | 17 | 0.0071882 | 0.02374 | 4.31 | 99.66 | 0.215 | Age(mean) | 6 | -0.0012013 | 0.01708 | -24.74 | 99.18 | 0.915 |
| BMI (mean) | 15 | -0.0423114 | 0.01654 | 6.12 | 99.29 | 0.189 | BMI (mean) | 5 | 0.0973748 | 0.00616 | 44.76 | 94.18 | 0.138 |
| Tobacco use (%) | 15 | 0.0022872 | 0.02895 | -3.32 | 99.60 | 0.473 | Tobacco use (%) | 7 | 0.0032490 | 0.07360 | -15.37 | 99.42 | 0.669 |
| Alcohol use (%) | 11 | 0.0021501 | 0.02132 | 0.39 | 99.32 | 0.338 | Alcohol use (%) | 5 | 0.0029205 | 0.06640 | -18.93 | 99.14 | 0.583 |
| Gender (women%) | 18 | 0.0048756 | 0.02326 | 1.70 | 99.64 | 0.269 | Gender (women%) | 7 | 0.0044783 | 0.06835 | -14.50 | 99.77 | 0.642 |
| Education (primary %) | 10 | 0.0017691 | 0.02341 | -0.70 | 99.66 | 0.359 | Education (primary %) | 6 | 0.0031054 | 0.04432 | 0.54 | 98.84 | 0.366 |
| Residence(urban%) | 7 | 0.0011579 | 0.03451 | -18.97 | 99.80 | 0.841 | Residence(urban%) | 3 | -0.0247619 | 0.00000 | 100.00 | 0.00 | 0.048 |
| Study year | 23 |  | 0.02458 | -2.36 | 99.65 |  | Study year | 9 |  | 0.06531 | -8.28 | 99.68 |  |
| <2010 | 5 | 0.0568445 |  |  |  | 0.488 | >=2010 | 8 | 0.1707090 |  |  |  | 0.550 |
| >=2010(ref) | 18 |  |  |  |  |  | <2010(ref) | 1 |  |  |  |  |  |
| Variable | Treatment | | | | | | Variable | Control | | | | | |
|  | N | Coefficient | Tau^2^ | Adj.R^2^(%) | *I^2^* | *P* |  | N | Coefficient | Tau^2^ | Adj.R^2^(%) | *I^2^* | *P* |
| Block 0 | 9 | 0.2237012 | 0.02703 |  | 98.95 | 0.004 | Block 0 | 9 | 0.0912732 | 0.00993 |  | 98.37 | 0.027 |
| Age(mean) | 6 | -0.0114276 | 0.01025 | 16.71 | 98.02 | 0.233 | Age(mean) | 6 | -0.0035396 | 0.00063 | 31.24 | 90.24 | 0.159 |
| BMI (mean) | 5 | 0.1237279 | 0.00493 | 65.52 | 96.31 | 0.064 | BMI (mean) | 5 | 0.0362585 | 0.00036 | 68.58 | 88.84 | 0.061 |
| Tobacco use (%) | 7 | 0.0032607 | 0.04061 | -11.68 | 98.55 | 0.568 | Tobacco use (%) | 7 | 0.0015496 | 0.01425 | -14.84 | 98.32 | 0.644 |
| Alcohol use (%) | 5 | 0.0034275 | 0.06184 | -12.82 | 99.44 | 0.510 | Alcohol use (%) | 5 | 0.0012107 | 0.00588 | -10.41 | 97.72 | 0.465 |
| Gender (women%) | 7 | -0.0049487 | 0.02910 | -5.57 | 98.96 | 0.444 | Gender (women%) | 7 | 0.0018033 | 0.01361 | -16.13 | 98.80 | 0.675 |
| Education (primary %) | 6 | 0.0017652 | 0.05116 | -17.18 | 99.20 | 0.618 | Education (primary %) | 6 | 0.0005632 | 0.00502 | -21.94 | 95.63 | 0.621 |
| Residence(urban%) | 3 | -0.0020635 | 0.00000 | 0.00 | 0.00 | 0.433 | Residence(urban%) | 3 | -0.0107197 | 0.02884 | -41.83 | 99.29 | 0.634 |
| Study year | 9 |  | 0.02456 | 9.13 | 98.1 |  | Study year | 9 |  | 0.01013 | -2.06 | 97.88 |  |
| >=2010 | 8 | 0.2225481 |  |  |  | 0.226 | >=2010 | 8 | 0.0995157 |  |  |  | 0.385 |
| <2010(ref) | 1 |  |  |  |  |  | <2010(ref) | 1 |  |  |  |  |  |

**Table 4 Meta-regression analysis of the Yi group to explore potential sources of heterogeneity, prevalence**

| Variable | N | Coefficient | Tau^2^ | Adj.R^2^(%) | *I^2^* | *P* |
| --- | --- | --- | --- | --- | --- | --- |
| Block 0 | 7 | 0.2800153 | 0.01725 |  | 99.53 | 0.001 |
| Age (mean) | 5 | 0.0116382 | 0.02113 | -12.94 | 99.50 | 0.514 |
| BMI (mean) | 5 | 0.0457334 | 0.00436 | 76.68 | 97.75 | 0.034 |
| Gender (women%) | 5 | -0.0018455 | 0.02321 | -24.05 | 99.58 | 0.665 |
| Residence (urban%) | 5 | 0.0116382 | 0.02113 | -12.94 | 99.50 | 0.514 |
| Study year |  |  | 0.00712 | 57.40 | 94.19 |  |
| >=2010 | 2 | 0.2091233 |  |  |  | 0.036 |
| <2010(ref) | 5 |  |  |  |  |  |

**Supplementary figures 1-4. The graphs of sensitivity analyses**

1A prevalence of Han


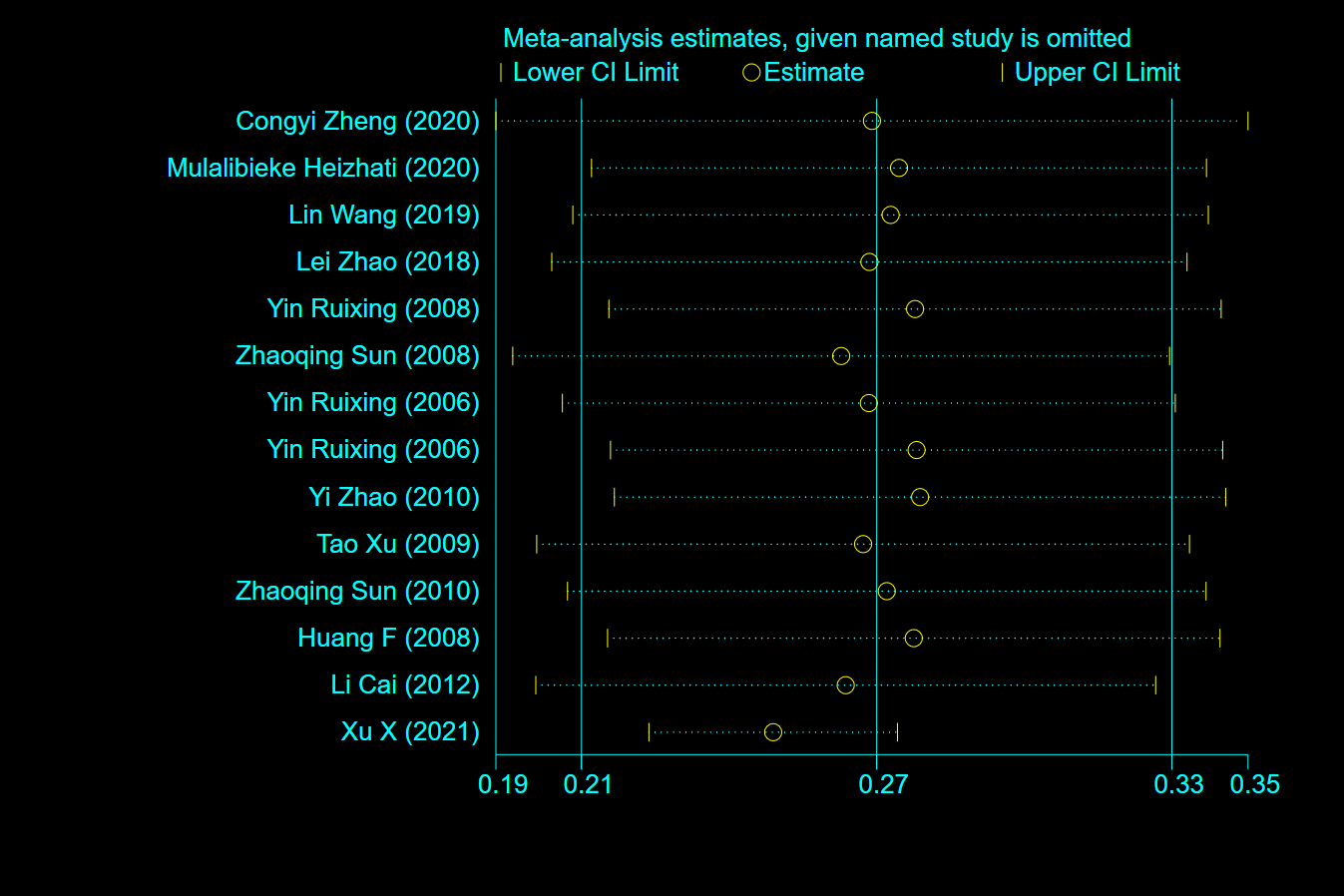


1B awareness of Han


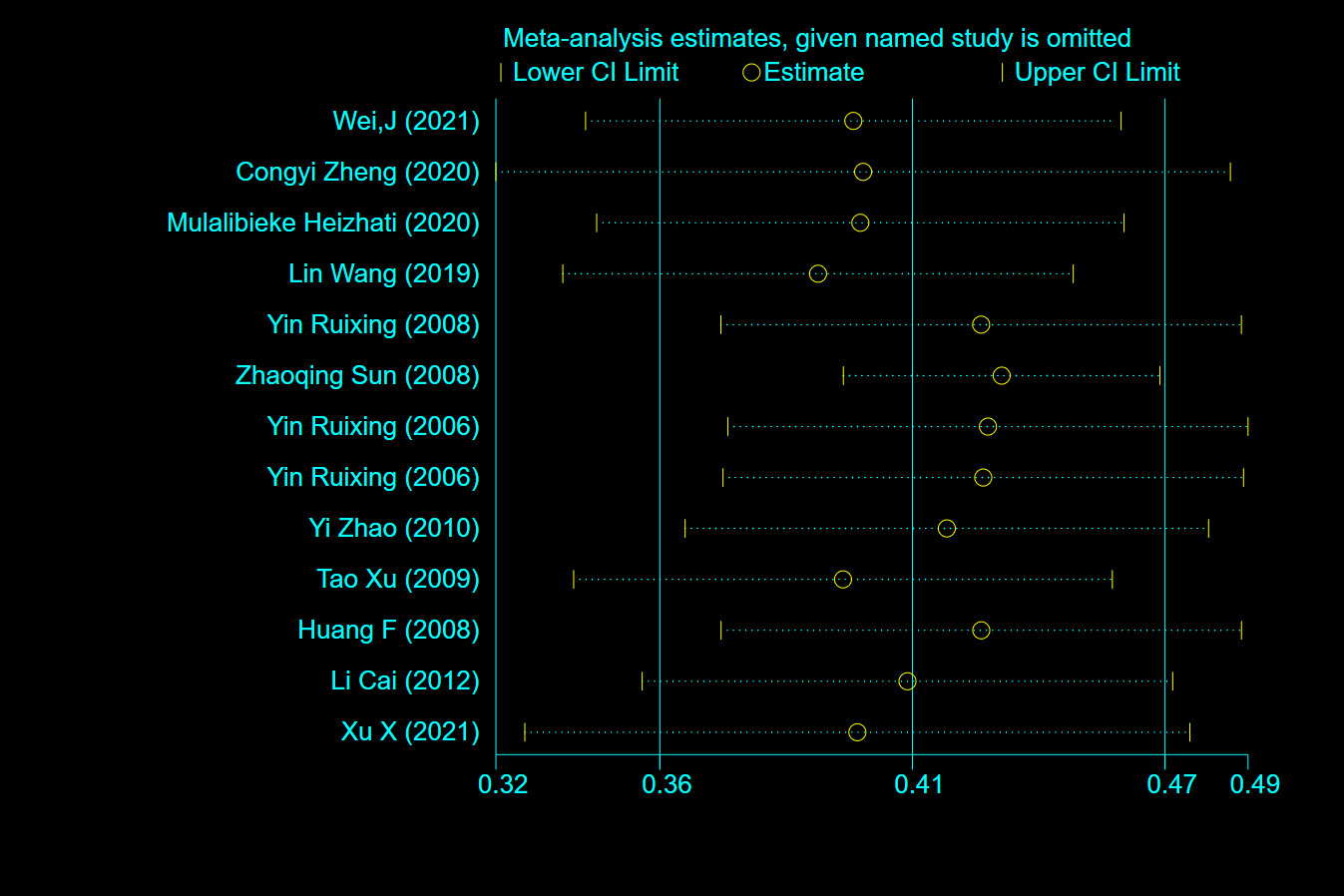


1C treatment of Han


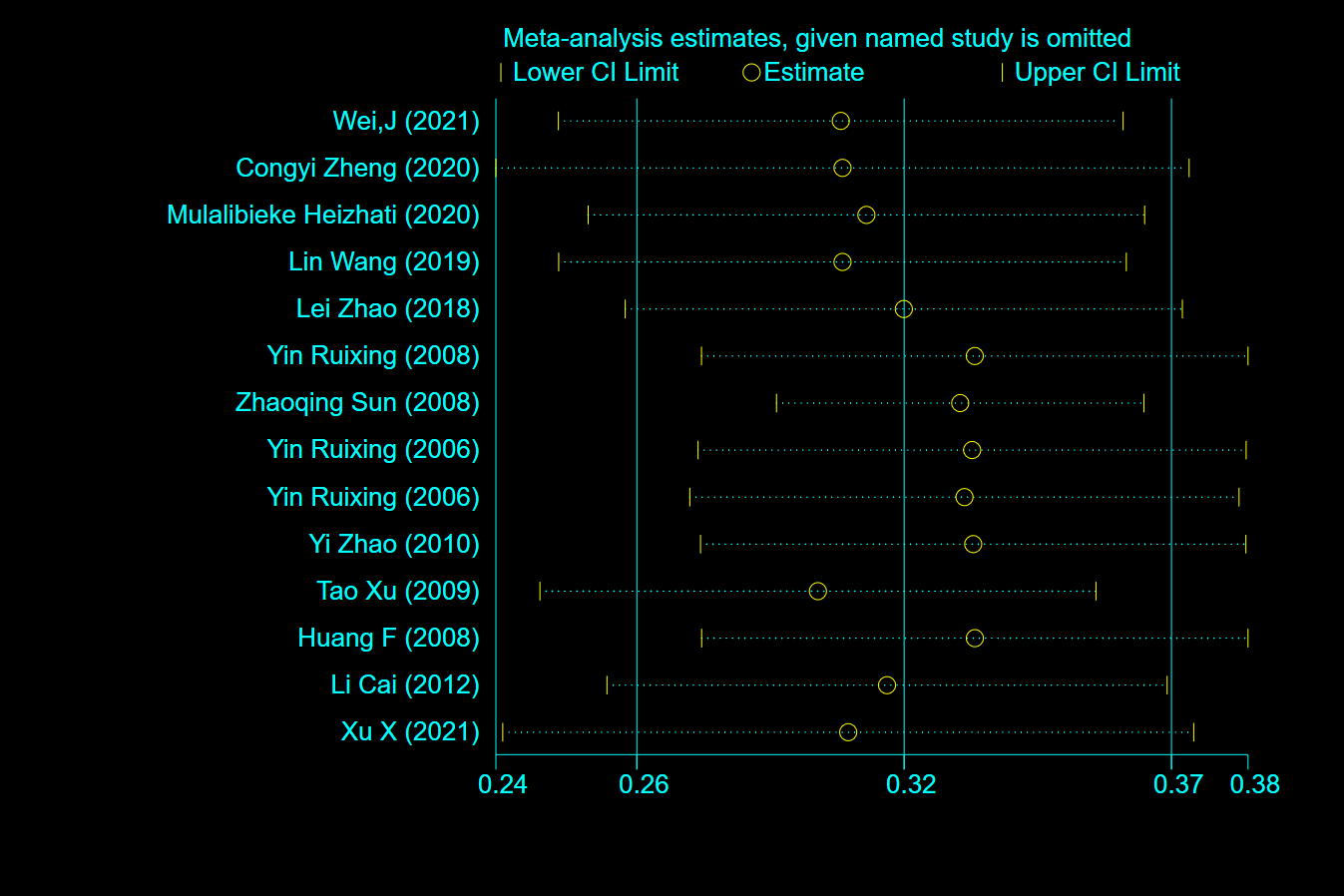


1D control of Han


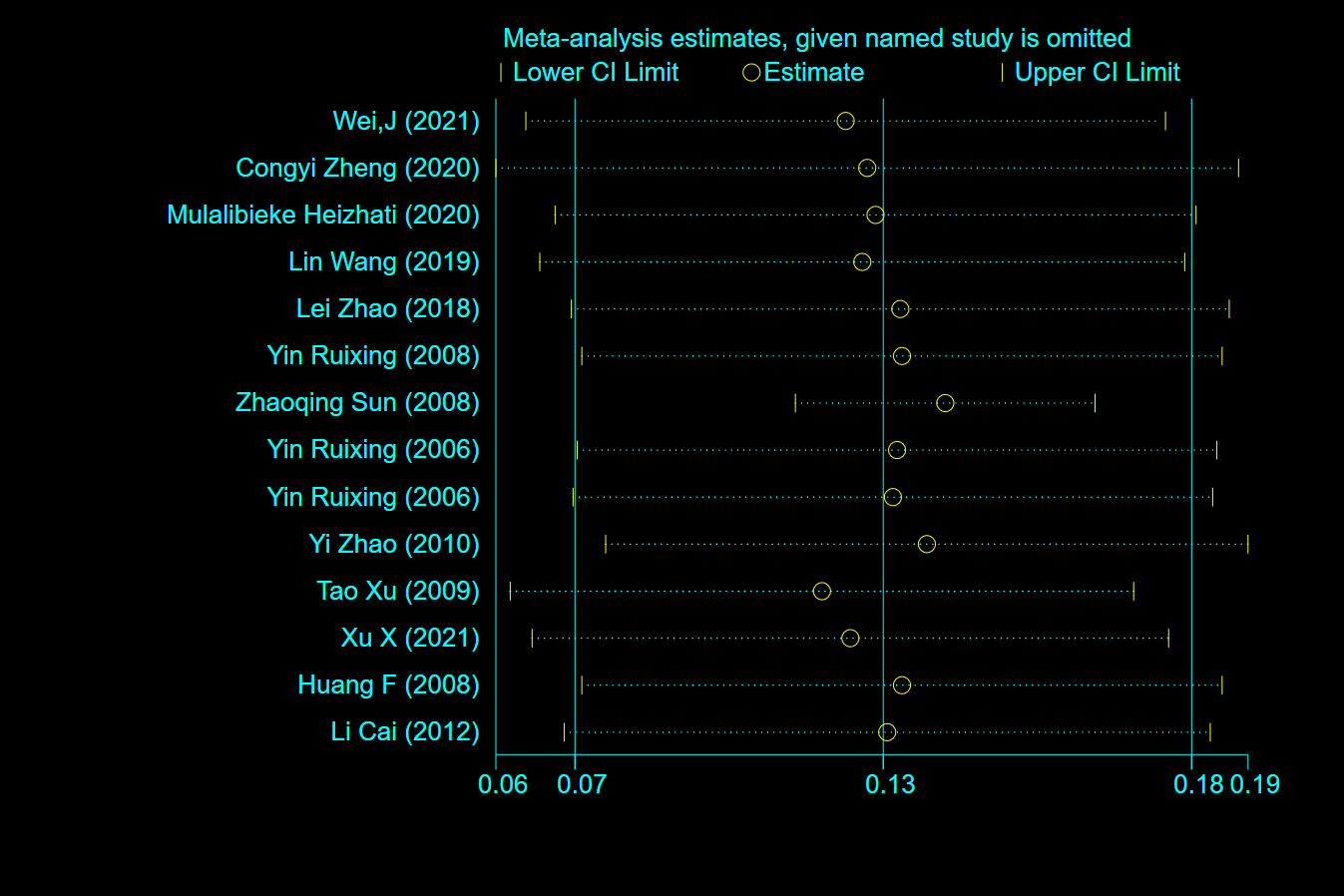


**Figure 1. the sensitivity analyses of data from the Han population**

2A prevalence of Tibetan


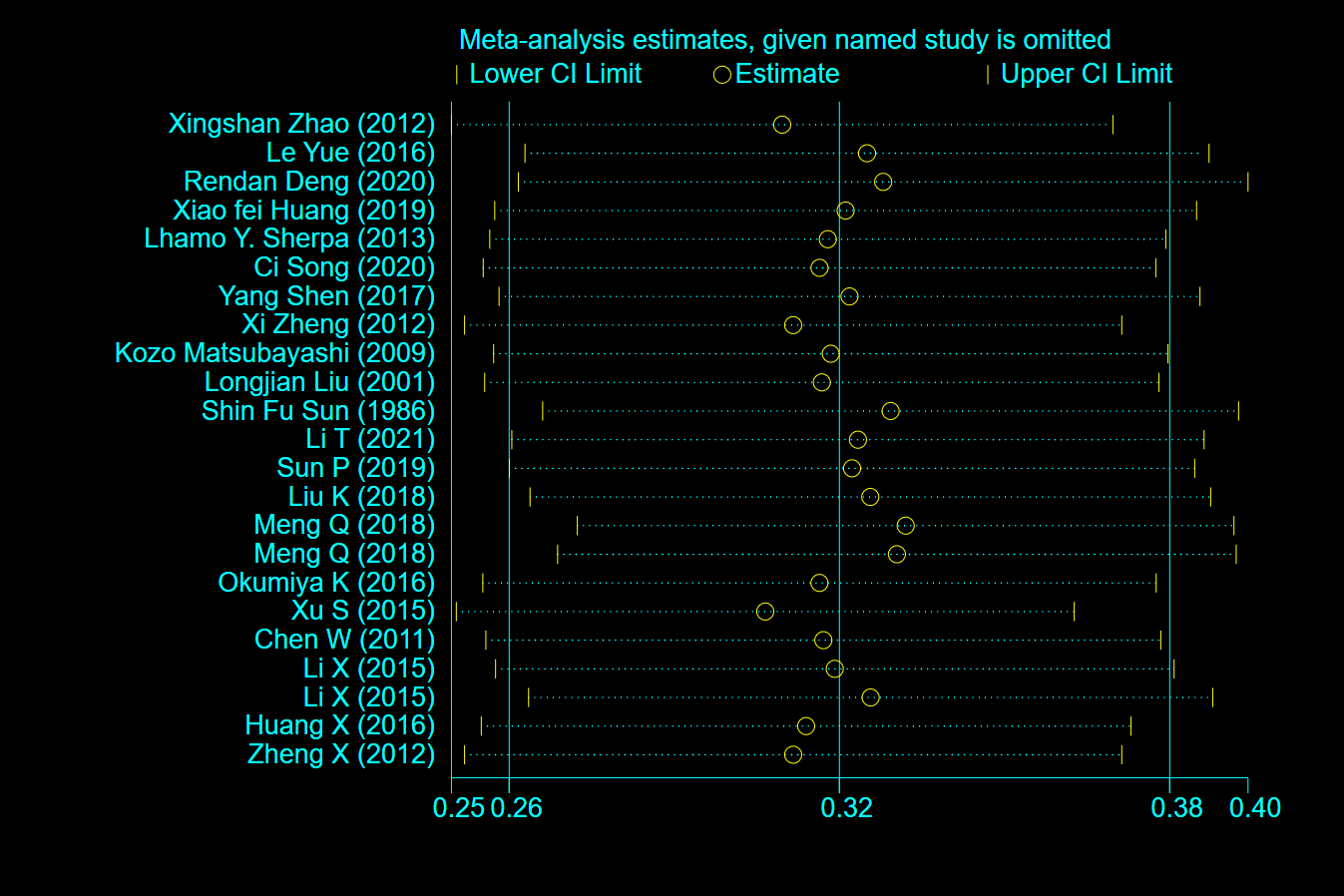


2B awareness of Tibetan


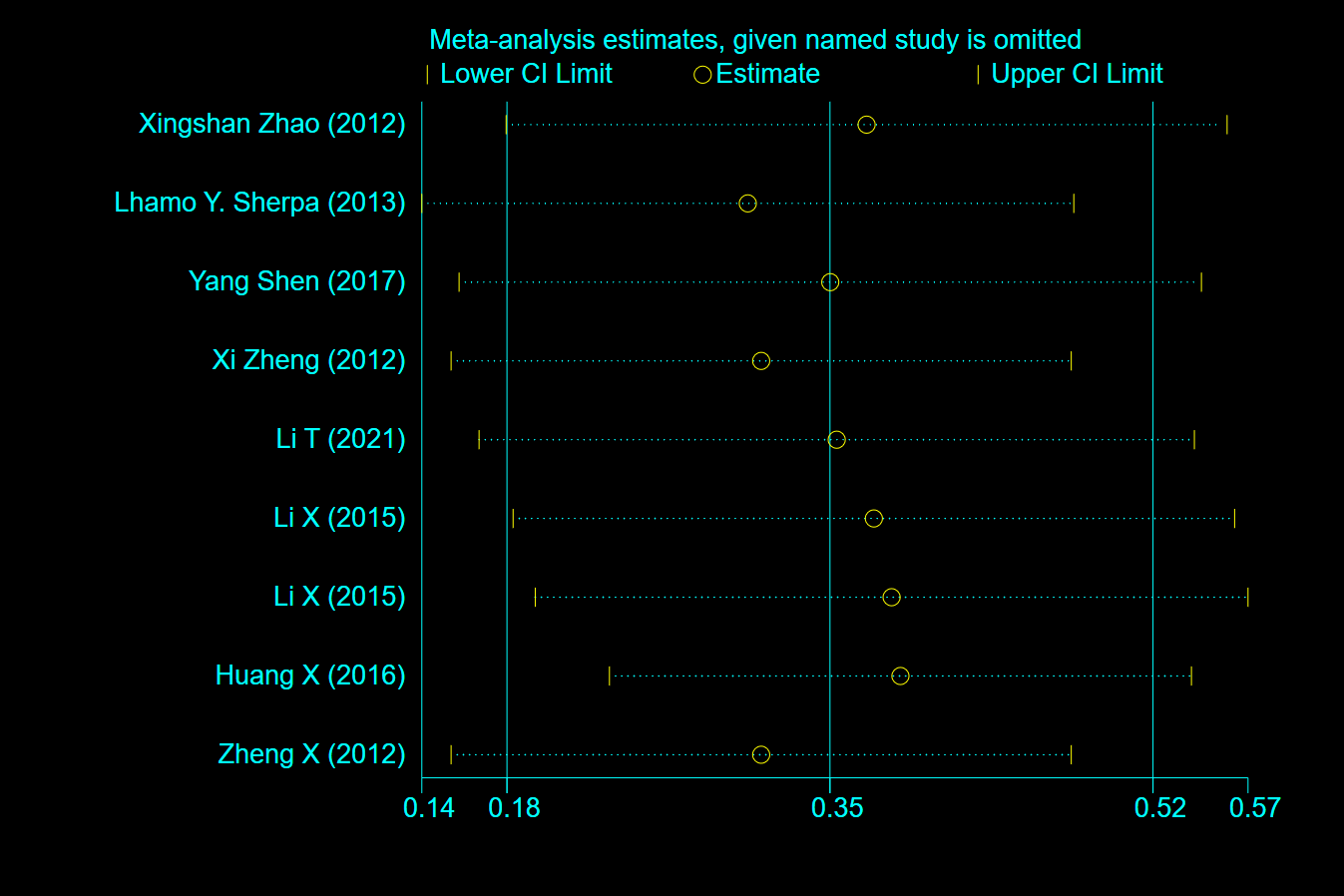


2C treatment of Tibetan


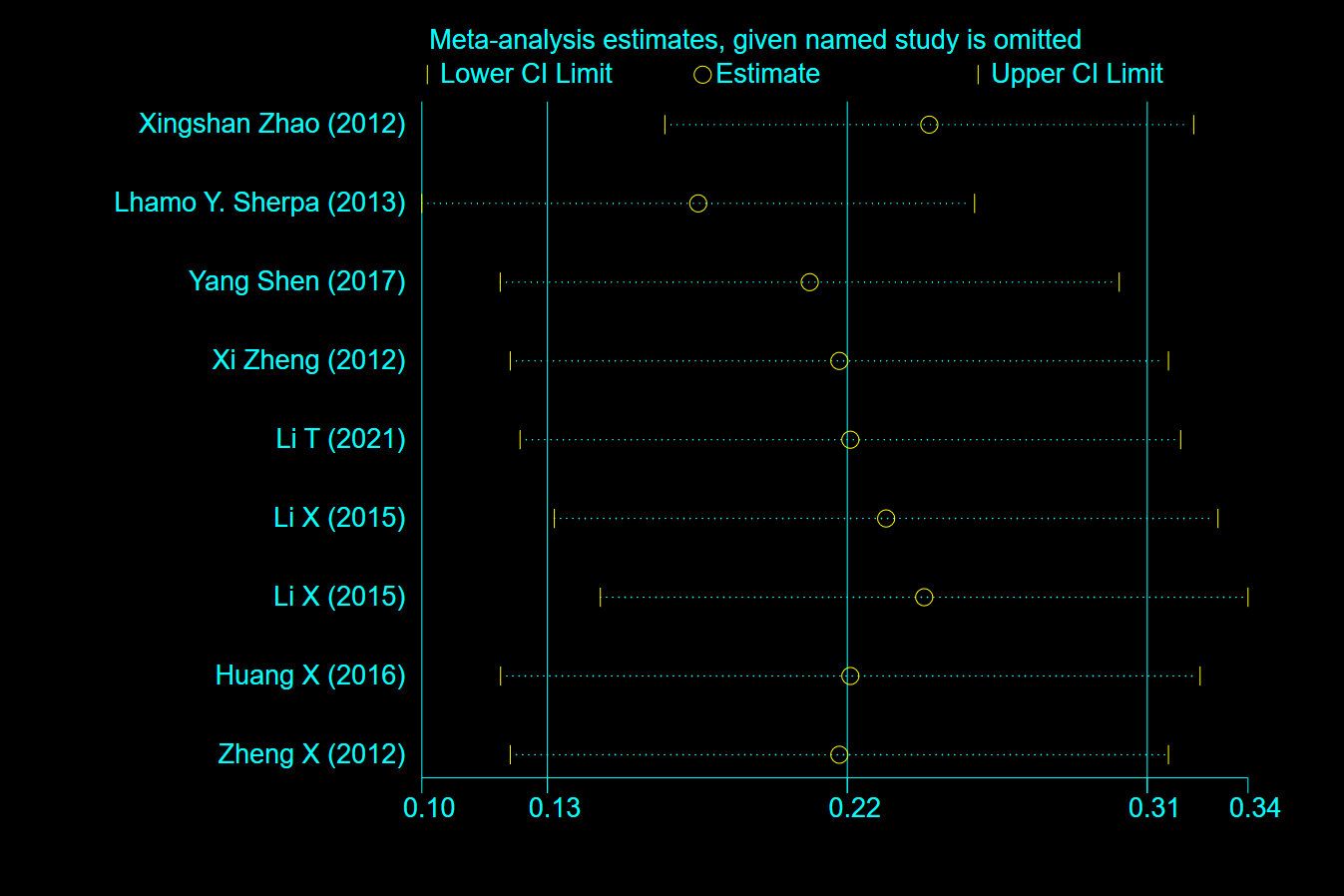


2D control of Tibetan


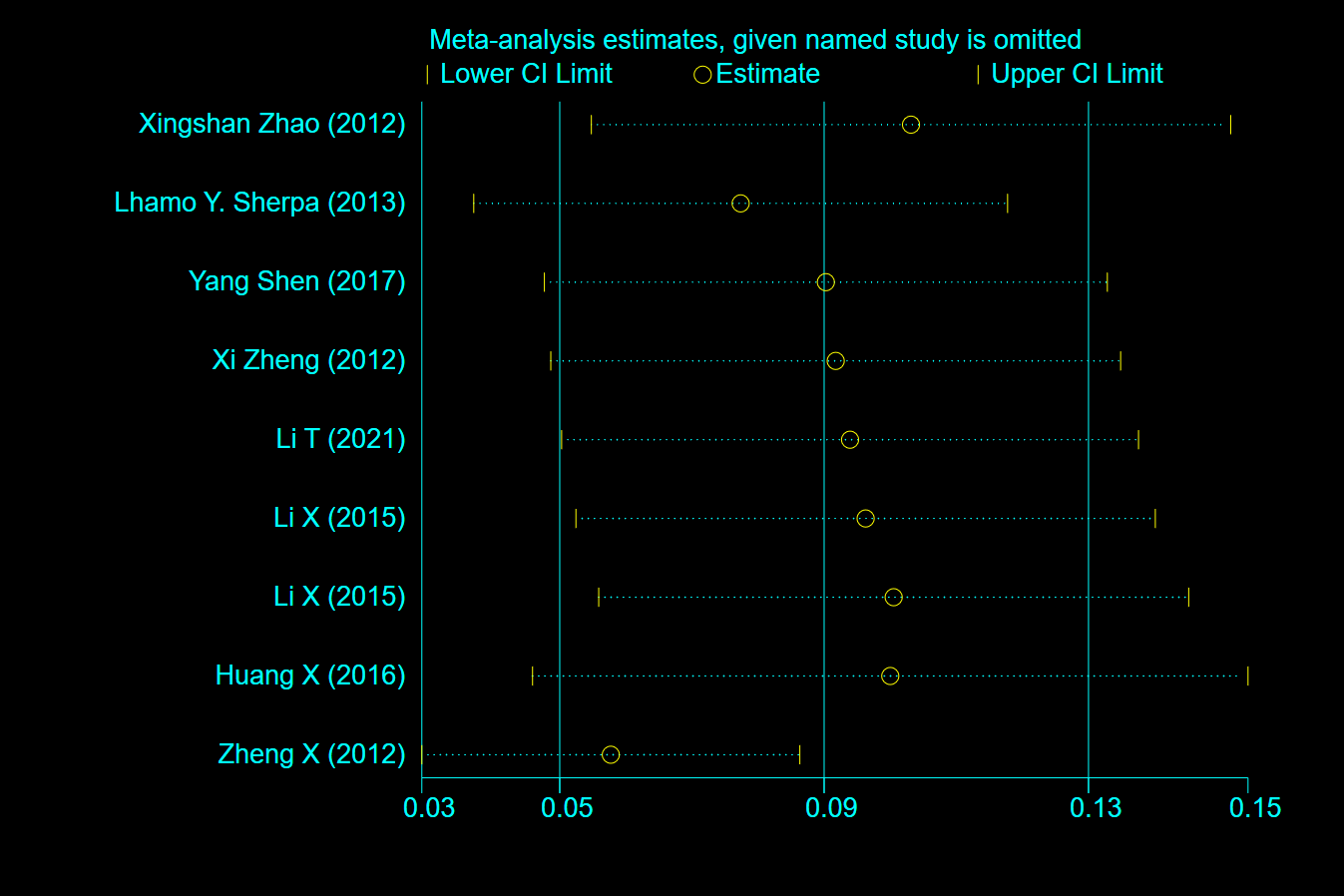


**Figure 2. the sensitivity analyses of data from the Tibetan population**

3A prevalence of Yi


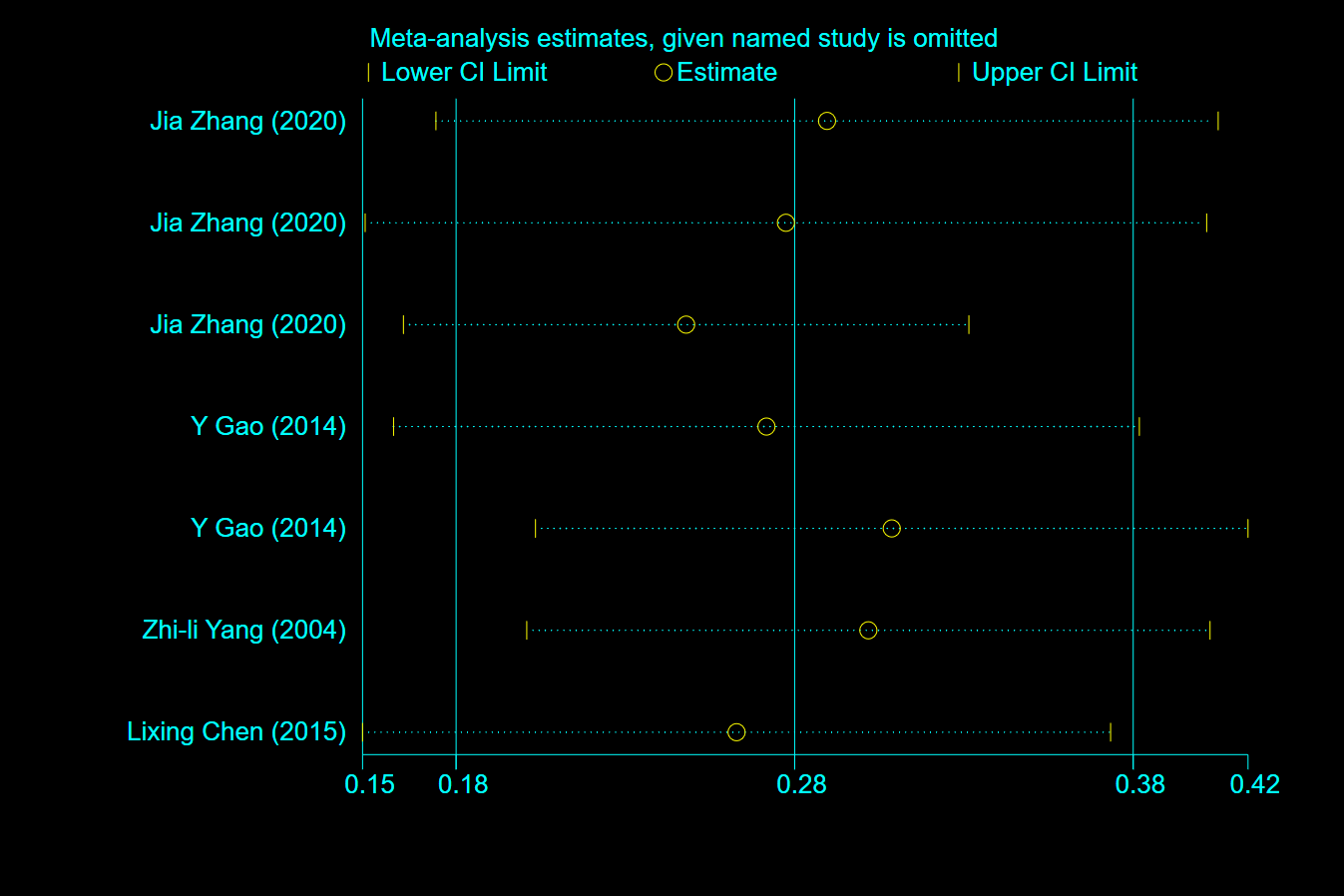


3B awareness of Yi


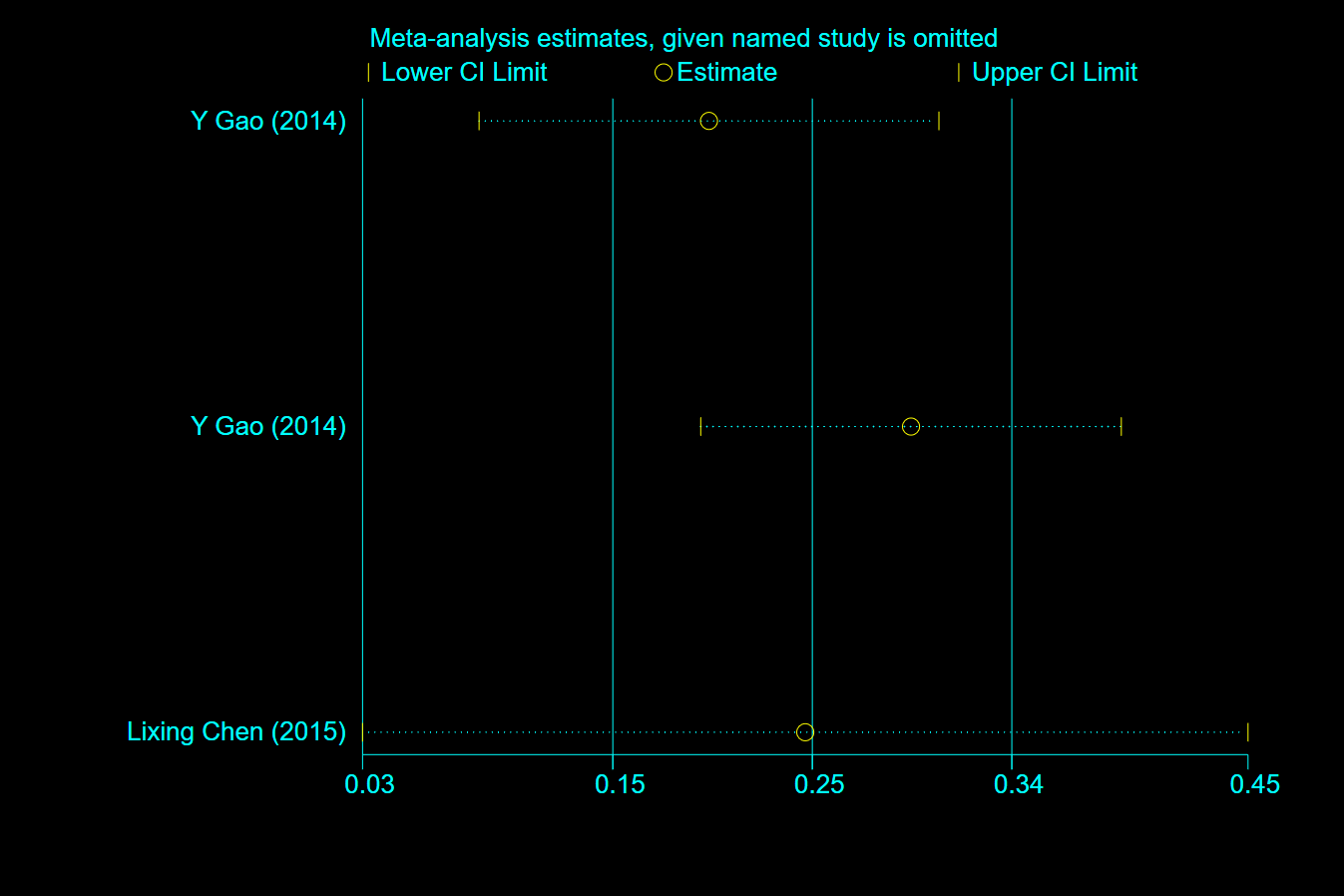


3C treatment of Yi


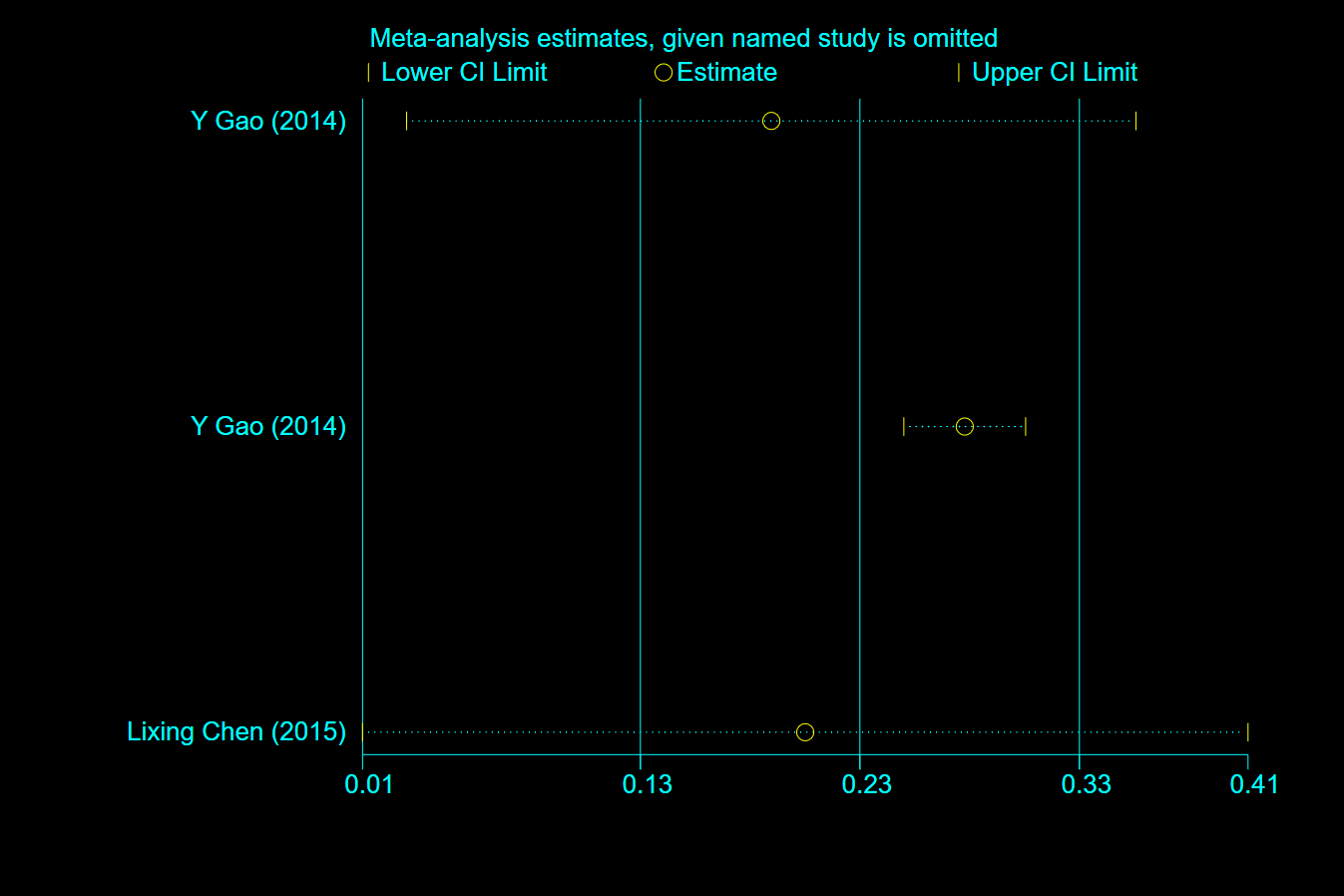


3D Control of Yi

**
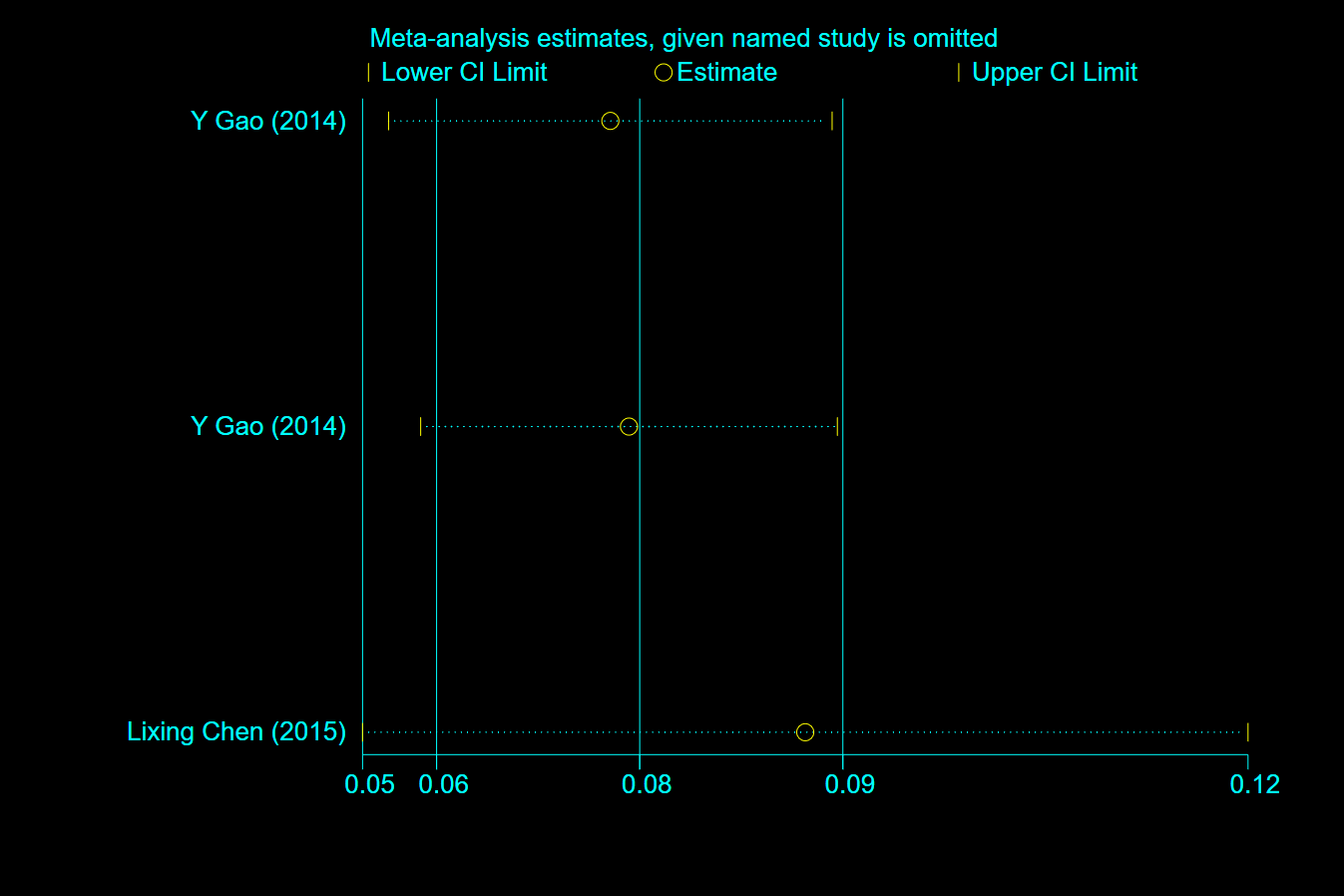
**

**Figure 3. the sensitivity analyses of data from the Yi population**

4A prevalence of Mongolian


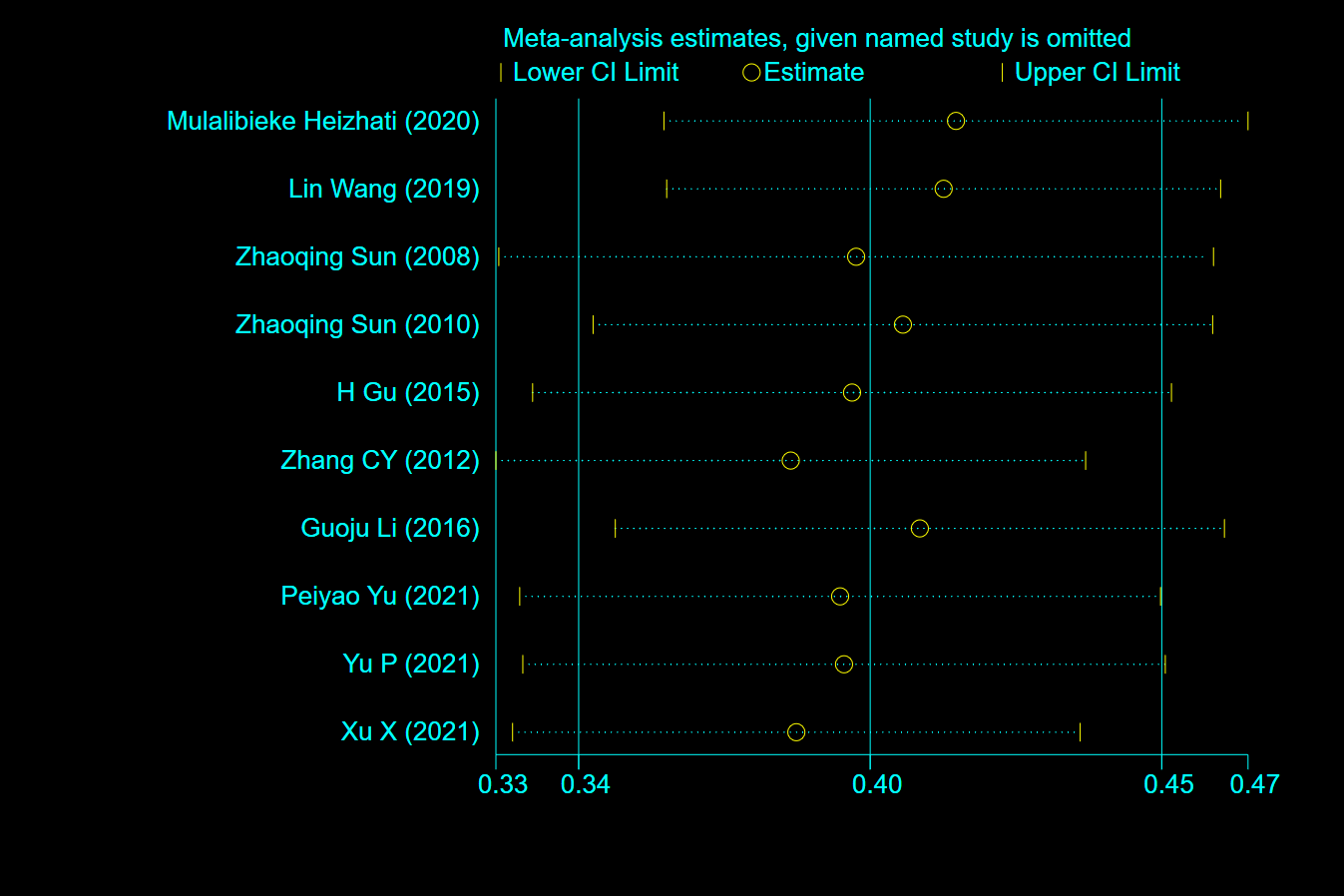


4B awareness of Mongolian


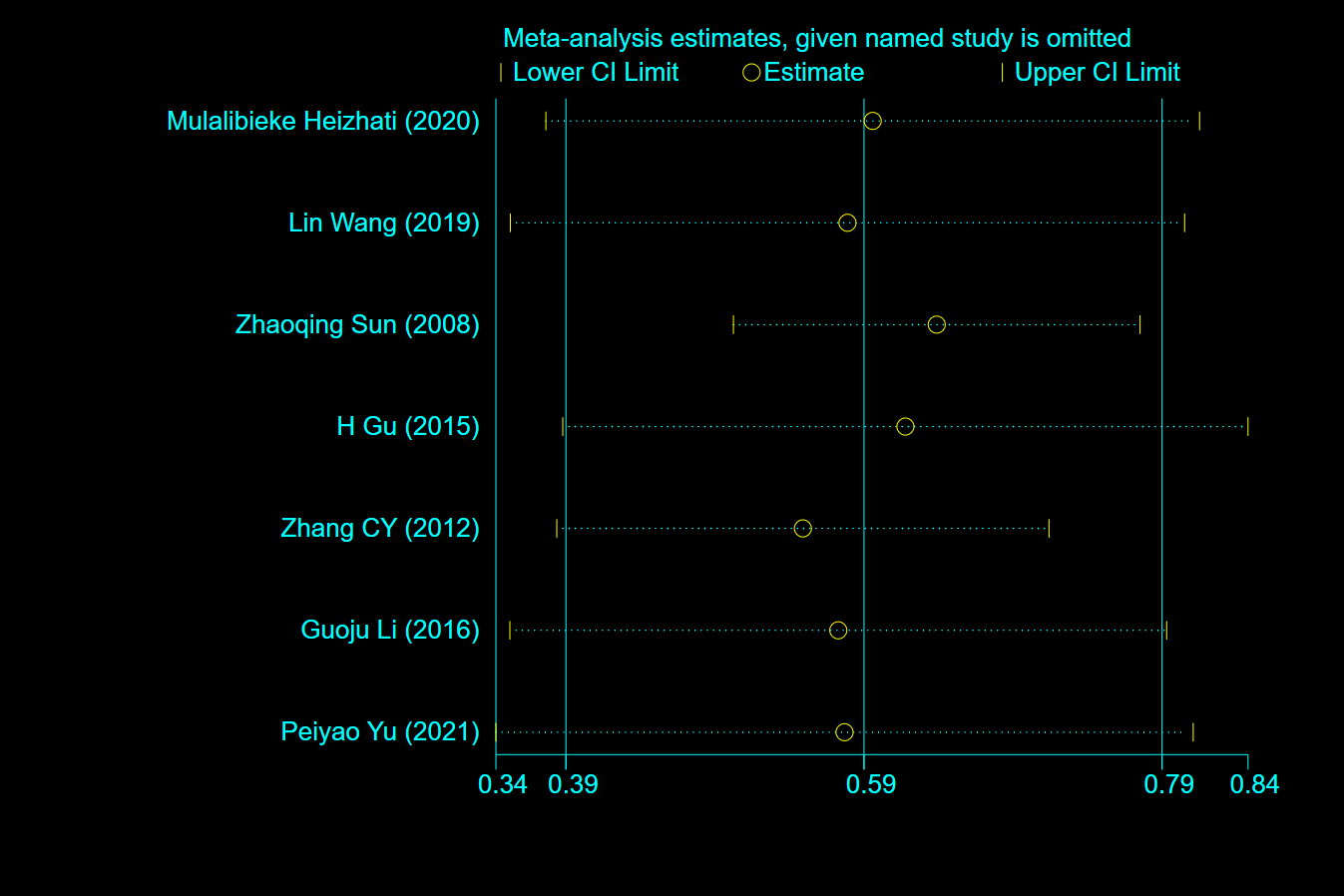


4C treatment of Mongolian


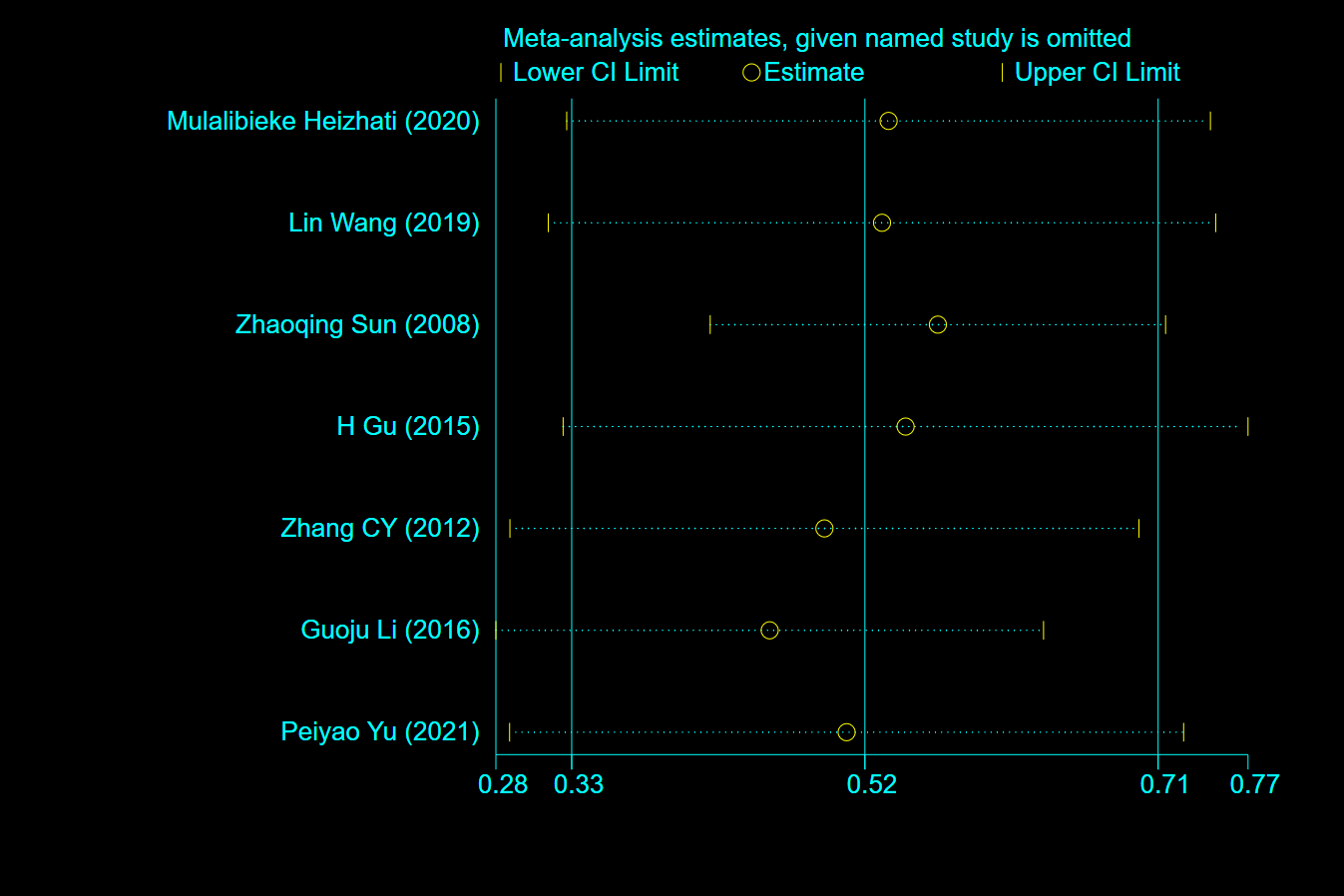


4D control of Mongolian

**
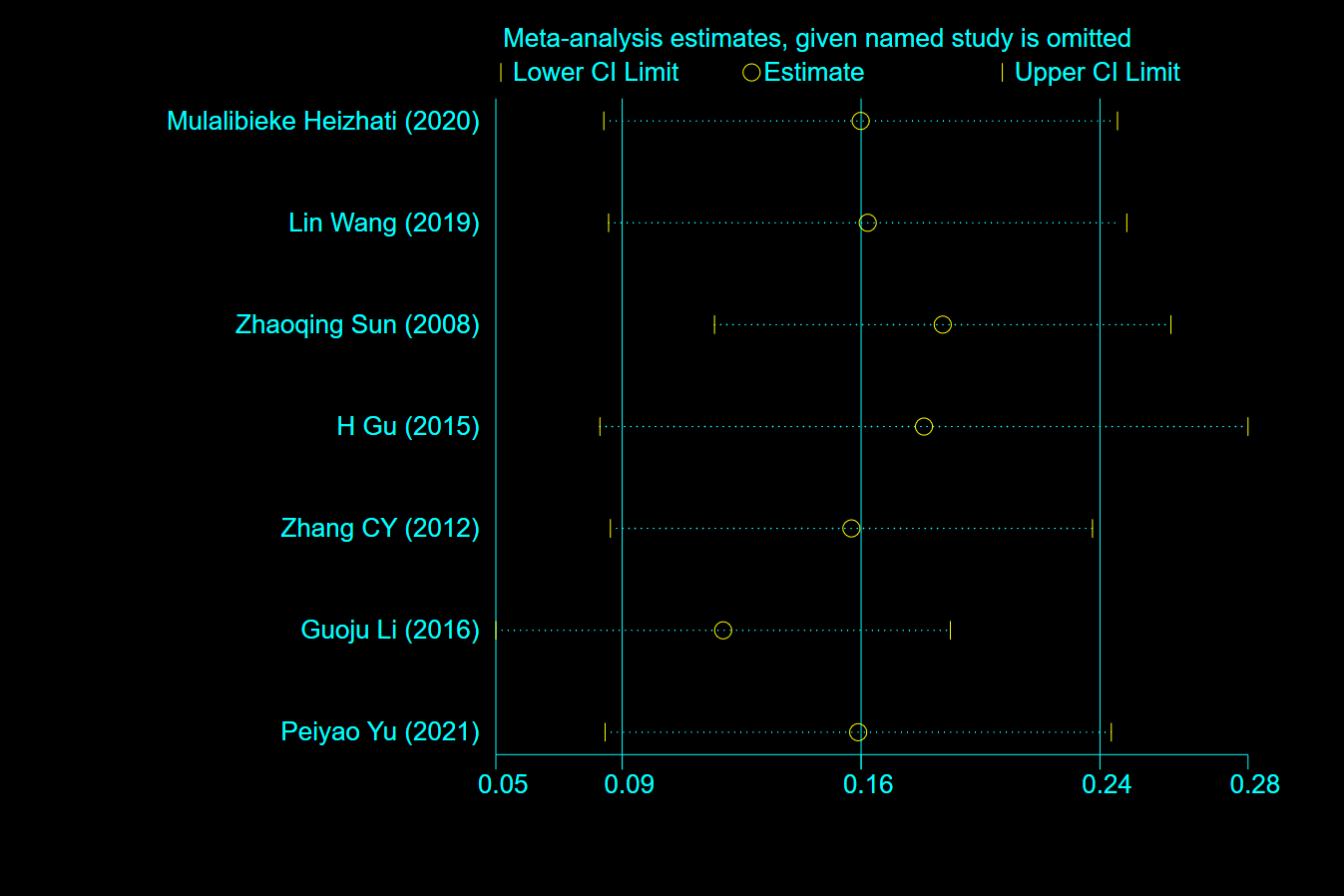
**

**Figure 4.** **the sensitivity analyses of data from the Mongolian population**
